# Adaptation on xylose improves glucose–xylose co-utilization and ethanol production in a carbon catabolite repression (CCR) compromised ethanologenic strain

**DOI:** 10.1186/s12934-022-01879-1

**Published:** 2022-08-06

**Authors:** Chandra Dev, Syed Bilal Jilani, Syed Shams Yazdani

**Affiliations:** 1 Microbial Engineering Group, International Centre for Genetic Engineering and Biotechnology, New Delhi, India; 2 DBT-ICGEB Centre for Advanced Bioenergy Research, International Centre for Genetic Engineering and Biotechnology, New Delhi, India

**Keywords:** *E. coli* B, Ethanologenic strain, EIIBC^Glc^, Sugar mixture, Co-utilization, Bioreactor, Carbon catabolite repression, Adaptive laboratory evolution

## Abstract

**Background:**

Sugar hydrolysates from lignocellulosic biomass are majorly composed of glucose and xylose that can be fermented to biofuels. Bacteria, despite having the natural ability to consume xylose are unable to consume it in presence of glucose due to a carbon catabolite repression (CCR) mechanism. This leads to overall reduced productivity as well as incomplete xylose utilization due to ethanol build-up from glucose utilization. In our effort to develop a strain for simultaneous fermentation of glucose and xylose into ethanol, we deleted *ptsG* in ethanologenic *E. coli* SSK42 to make it deficient in CCR and performed adaptive laboratory evolution to achieve accelerated growth rate, sugar consumption and ethanol production. Finally, we performed proteomics study to identify changes that might have been responsible for the observed improved phenotype of the evolved strain.

**Results:**

The parental strain of SSK42, i.e., wild-type *E. coli* B, did not co-utilize glucose and xylose as expected. After deleting the *ptsG* gene encoding the EIIBC^Glc^ subunit of PTS system, glucose consumption is severely affected in wild-type *E. coli* B. However, the ethanologenic, SSK42 strain, which was evolved in our earlier study on both glucose and xylose, didn’t show such a drastic effect of EIIBC^Glc^ deletion, instead consumed glucose first, followed by xylose without delay for switching from one sugar to another. To improve growth on xylose and co-utilization capabilities, the *ptsG* deleted SSK42 was evolved on xylose. The strain evolved for 78 generations, strain SCD78, displayed significant co-utilization of glucose and xylose sugars. At the bioreactor level, the strain SCD78 produced 3-times the ethanol titer of the parent strain with significant glucose–xylose co-utilization. The rate of glucose and xylose consumption also increased 3.4-fold and 3-fold, respectively. Proteome data indicates significant upregulation of TCA cycle proteins, respiration-related proteins, and some transporters, which may have a role in increasing the total sugar consumption and co-utilization of sugars.

**Conclusion:**

Through adaptive evolution, we have obtained a strain that has a significant glucose–xylose co-utilization phenotype with 3-fold higher total sugar consumption rate and ethanol production rate compared to the unevolved strain. This study also points out that adaptation on xylose is enough to impart glucose–xylose co-utilization property in CCR compromised ethanologenic strain SSK42.

**Supplementary Information:**

The online version contains supplementary material available at 10.1186/s12934-022-01879-1.

## Background

The world is facing severe environmental problems due to excessive dependence on fossil fuels and their exhaustive use. Biofuels, especially those derived from lignocellulosic biomass, are considered as preferred option for producing energy because they are sustainable, eco-friendly, and do not compete with food [1]. A huge amount of lignocellulosic biomass waste (billions of tons) is generated annually worldwide, most of which is either burned or discarded [2]. The lignocellulosic biomass generally consists of 40–50% cellulose, 25–30% hemicellulose, 15–20% lignin and other minor components [3, 4]. Biomass can be biologically converted into bioethanol and other economically important products such as lactate, butanol, bioplastic etc. [5–10].

The biomass, after pretreatment and hydrolysis, is left with mainly the mixture of sugars. Glucose and xylose are the two major sugars in this mixture, accounting for ~ 90% of all the sugars [11]. The bacteria, like *E. coli*, has inherent capability of consuming hexoses as well as pentoses, as opposed to commonly used ethanol fermenting yeast, *Saccharomyces cerevisiae*, which naturally doesn’t have the ability to consume pentose sugars. Therefore, many efforts have been made to engineer these bacteria for ethanol production [12]. However, owing to the phenomena of carbon catabolite repression (CCR), the bacteria are only able to consume xylose after they have exploited all the glucose. This preferential sugar consumption strategy increases the total fermentation time and hence the economic burden on product formation at pilot scale. Simultaneous sugar consumption from a mixture of sugars (lignocellulosic biomass hydrolysate) is an efficient strategy for economical product formation [13].

To break the sequential sugar consumption phenotype, many strategies, including algorithm-based target identification [14] and ALE (Adaptive Laboratory Evolution) based sugar transporter engineering [16] have been adopted. However, the most widely exploited system for preventing the sequential sugar consumption had been the phosphotransferase system of glucose transport [17–19]. Zhou et al. have reported an increase in lactic acid production in *E. coli* strain devoid of glucose repression by deleting *ptsG* gene and performing ALE for improved glucose and xylose consumption [18]. After adaptation on glucose the average sugar consumption of 1.04 g/L/h was reported, nevertheless, xylose consumption remained minimal until glucose concentration fell below certain level. Zhao et al., also made use of a *ptsG* knockout *E. coli* strain for the production of valuable aromatic chemical 4-hydroxymandelic acid using a mixture of glucose and xylose [19]. Alterthum and Ingram have reported high ethanol production in *E. coli* using single substrate by heterologous expression of alcohol dehydrogenase and pyruvate decarboxylase from *Zymomonas mobilis* [12]. The ethanol titers from glucose and xylose were reported to be 58 g/L and 42 g/L, respectively. Nichols et al. have expressed these two enzymes in a CCR compromised (i.e., *ptsG* deleted) *E. coli* strain and demonstrated co-consumption of Glucose, xylose and arabinose for production of 4.15% (i.e., 32.7 g/L) ethanol [20]. However, the total fermentation time increased from 45 h in parental strain to 70 h in CCR compromised strain.

**Fig. 1:**
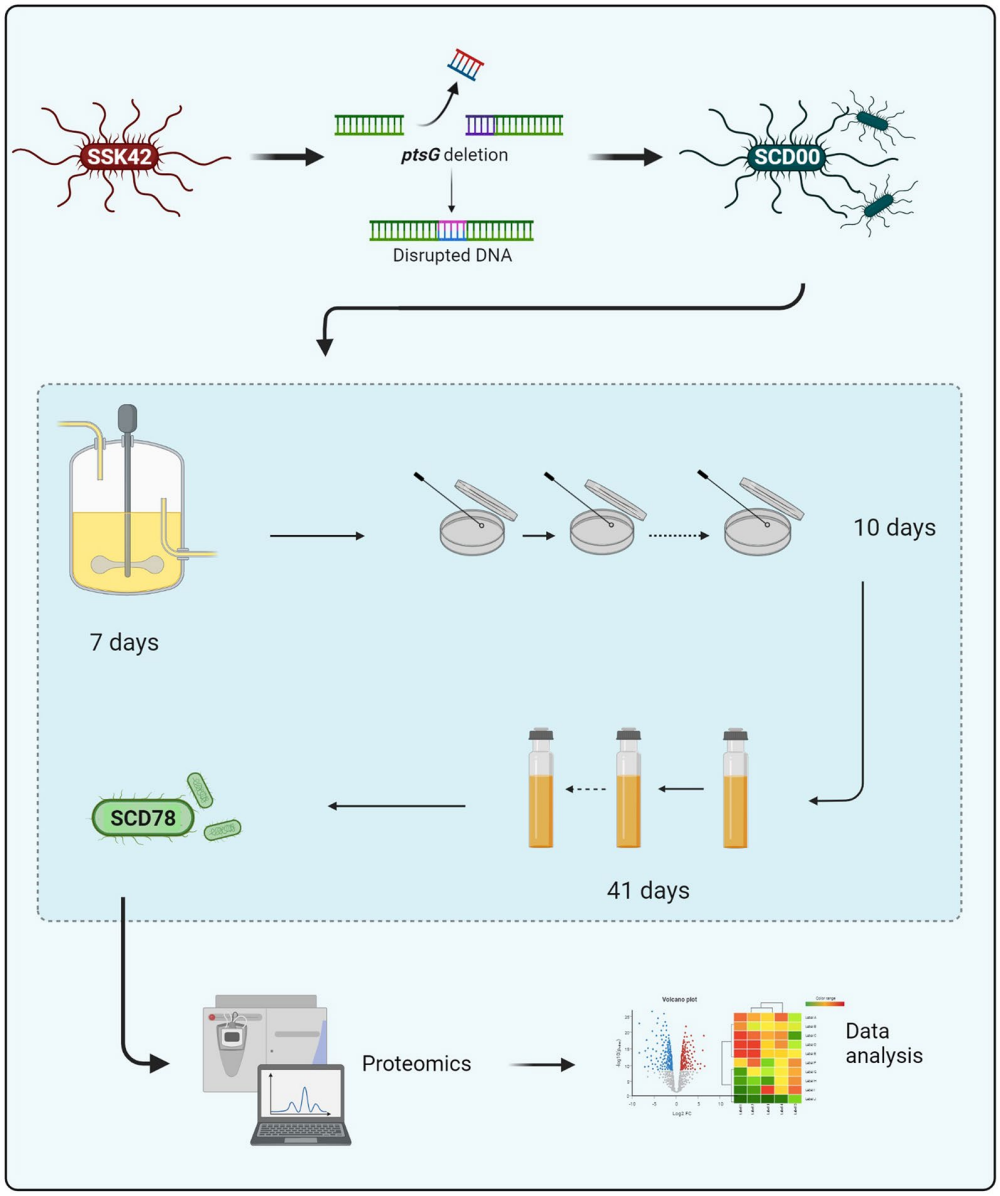
Schematic representation of the current study that includes strain engineering, adaptive laboratory evolution (ALE), and proteomics. ALE experiment consists of several steps of adaptation which include culture passaging in CSTR, on agar plates, and in Hungate tubes

In the current study, we wanted to address the issue of growth delay in the CCR compromised ethanologenic strain for co-fermentation of hexose and pentose sugars. We used *E. coli* strain SSK42, a derivative of *E. coli* B, as a platform host for this study as this strain was engineered earlier to produce ethanol without the need of any heterologous genes by modulating the expression of endogenous pathway [21, 22]. The strain SSK42 was developed by deleting the genes for competing pathways for ethanol production i.e., Δ*ldhA, ΔfrdA* and Δ*pflB*, and replacing the promoter of pyruvate dehydrogenase with *gapA* promoter for constitutive expression of the pyruvate dehydrogenase under anaerobic condition. The strain was further evolved alternatively on glucose and xylose to improve the growth rate and ethanol productivity on both the sugars [21, 22]. In this study, the *ptsG* gene was deleted in SSK42 to remove the CCR, and the adaptive laboratory evolution was performed to improve the growth and sugar consumption rate of the engineered strain (Fig. 1). Finally, the proteomic study was performed to understand the factors that might have contributed to the enhanced growth and sugar consumption.

## Results

### Effect of ***ptsG*** deletion on ***E. coli*** B and ethanologenic SSK42 strain

The *ptsG* gene was deleted in the parental *E. coli* B strain and its ethanologenic derivative SSK42 (Table 1) to evaluate and compare its deletion impact on CCR mechanism in these strains. The sugar consumption phenotype of all the strains was determined in minimal media containing a mixture of glucose and xylose (~ 1.5 g/L each sugar) as carbon source. Experiment was performed in screw capped Hungate tubes to maintain micro-aerobic conditions. Samples were removed from the tubes every three hours and analyzed for growth, sugars and metabolites, as mentioned in the methods section.

**Table 1:** Details of strains used in the study

S. No.	Strain	Description	Reference
1	*E. coli* B	Wild type	CGSC Catalog no. 2507
2	*E. coli* JW1087	Keio strain having *ptsG* deletion in BW25113; used for phage transduction in this study	CGSC Catalog no. 9031
3	*E. coli* B Δ*ptsG*	*ptsG* gene encoding EIIBC component of PTS deleted in *E. coli* B	This study
4	SSK42	*E. coli* B Δ*ldhA* Δ*frdA* Δ*pflB* Δ*PDH* promoter : : *gapA* gene promoter, further adapted on glucose and xylose to improve productivity	[22]
5	SCD00	*ptsG* gene encoding EIIBC component of PTS deleted in SSK42	This study
6	SCD78	SCD00 adapted on xylose to improve productivity	This study

**Fig. 2:**
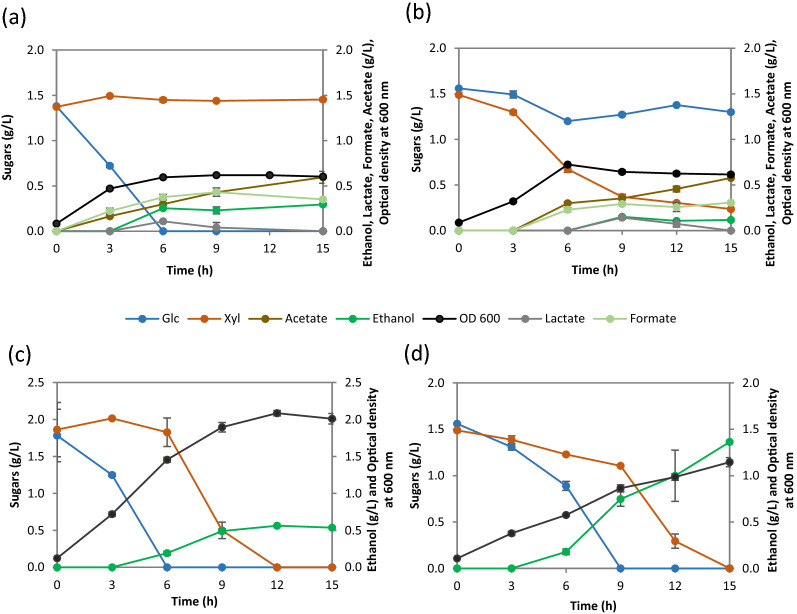
Sugar consumption, ethanol production and growth profiles of (**a**) *E. coli* B, (**b**) *E. coli* B Δ*ptsG*, (**c**) SSK42 and (**d**) SCD00 (i.e., SSK42 Δ*ptsG*) in Hungate tube experiments. The data represents the average of three biological replicates, and error bars represent the standard deviation

The wild type *E. coli* B strain consumed complete glucose within 6 h of growth, while no significant consumption of xylose was observed under the given condition (Fig. 2a). It was noticed that the strain *E. coli* B did not switch to xylose sugar even after 9 h of completely consuming the glucose. Upon *ptsG* deletion, the strain *E. coli* B Δ*ptsG* showed a severely impaired glucose metabolism with only ≈ 0.3 g/L glucose consumed in 15 h (Fig. 2b). This was not surprising because the most dominant glucose PTS transporter component EIIBC^Glc^ has been deleted in this strain. The elimination of the carbon catabolite repression due to deletion of *ptsG* permitted the cells to now consume ≈ 1.2 g/L of xylose in 15 h at a maximum consumption rate of 0.21 ± 0.02 g/L/h, with ≈ 0.3 g/L of xylose still remaining in the medium (Table 2; Fig. 2b).

**Table 2:** Sugar consumption and product formation profiles in the Hungate tube experiment

Parameters of sugar consumption, product formation, and growth during 15 h of cultivation				
Strains	Max. sugar consumption rate (g/L/h)		Rate of production (g/L/h)	
	Glucose (Time interval)	Xylose (Time interval)	Cells	Ethanol
*E. coli* B	0.24 ± 0.01 (3–6 h)	No consumption	0.06 ± 0.00	0.08 ± 0.00
*E. coli* B Δ*ptsG*	0.02 ± 0.00 (3–15 h)	0.21 ± 0.02 (3–6 h)	0.07 ± 0.00	0.05 ± 0.00
SSK42	0.42 ± 0.01 (3–6 h)	0.44 ± 0.03 (6–9 h)	0.12 ± 0.00	0.56 ± 0.00
SCD00	0.30 ± 0.02 (6–9 h)	0.27 ± 0.03 (9–12 h)	0.05 ± 0.01	0.19 ± 0.02
SCD78	0.22 ± 0.00 (6–9 h)	0.17 ± 0.01 (3–6 h)	0.06 ± 0.01	0.24 ± 0.00

The sugar utilization pattern of SSK42 and its Δ*ptsG* derivative (strain SCD00) was relatively better. Similar to *E. coli* B, the ethanologenic strain SSK42 consumed all the glucose in first 6 h (Fig. 2c). Nevertheless, it quickly shifted to xylose metabolism without any delay and consumed all the xylose in next 6 h (Fig. 2c), as against *E. coli* B that could not shift its metabolism even after 9 h of glucose consumption (Fig. 2a). This phenotype of SSK42 could be due to the fact that it has been adapted using the alternating sugar strategy where the sugar was exchanged (glucose to xylose and vice-versa) with each successive passage [22], which might have helped in switching the metabolism on alternate sugar quickly. However, there was no evidence of co-utilization of glucose and xylose in SSK42 (Fig. 2c). When we analyzed the impact of *ptsG* deletion in SSK42, we found that the resultant strain SCD00 did not show as drastic effect on glucose utilization as shown by the *E. coli* B Δ*ptsG*; instead, it still consumed the glucose first, followed by the xylose (Fig. 2d), similar to SSK42. This phenotype could also be attributed to the previous passaging of SSK42 strain on alternate sugar [22], which perhaps allowed glucose to get transported inside the cells via other routes besides the glucose specific PTS system. The important observation in the fermentation profile of SCD00 was some degree of co-utilization of xylose (~ 0.4 g/L) in presence of glucose within 9 h of cultivation (Fig. 2d).

**Fig. 3:**
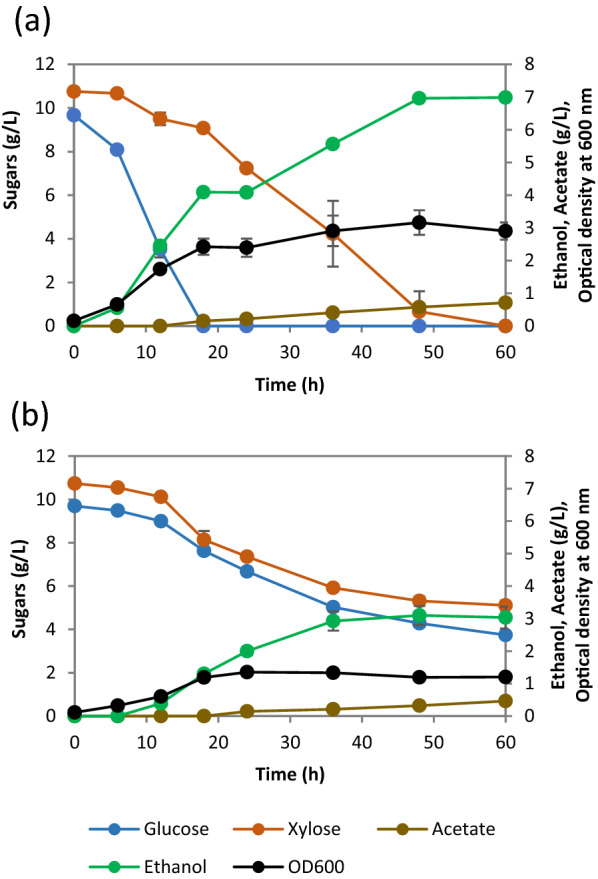
Sugar consumption, ethanol production, and growth profiles of (**a**) Strain SSK42 and (**b**) SCD00 in the bioreactor. Growth profile is represented by OD_600_. The data represents the average of two biological replicates, and error bars represent the standard deviation

To further confirm the co-utilization ability of SCD00, we cultivated the strain in a bioreactor under controlled conditions along with SSK42 as control at 10 g/L of each glucose and xylose. We observed in the bioreactor for the strain SSK42 that the glucose remained as preferred carbon source (Fig. 3a). It first consumed glucose in 18 h followed by additional 42 h for xylose consumption. It consumed all the sugar present in 60 h at an average total sugar consumption rate of 0.38 ± 0.07 g/L/h without significant co-utilization of the sugars. On the other hand, the fermentation profile of SCD00 indicated a significant co-utilization of both the sugars until about 36 h of cultivation (Fig. 3b), but took 124 h to consume glucose to completion while approximately 3.0 g/L xylose still remained in the media (Additional file 1: Fig. S1). The average total sugar consumption rate achieved by strain SCD00, i.e., 0.26 ± 0.01 g/L/h, was almost 1.6 times lesser than that of strain SSK42. The individual glucose and xylose consumption rates for strain SCD00 were 0.13 ± 0.00 g/L/h each, and for SSK42 were 0.54 ± 0.01 and 0.25 ± 0.00 g/L/h, respectively. This corresponded to roughly 4 fold decrease in glucose consumption rate and approximately 2 fold decrease in xylose consumption rate due to deletion of *ptsG* gene in SSK42. As a result of lower sugar consumption rate, the biomass and ethanol productivity also suffered significantly (Fig. 3a, b).

Strain SSK42 displayed almost continuous growth till both the sugars were consumed completely (Fig. 3a). On the other hand, the strain SCD00 displayed a biphasic growth pattern, where it exhibited co-utilization until the end of the first growth phase (0–36 h). The second growth phase (which starts at 60 h) appears to be primarily on glucose consumption, although xylose consumption was not completely stopped (Additional file 1: Fig. S1b). Strain SCD00 could only achieve a maximum optical density at 600 nm (OD_600_) of ~ 2 in 96 h as against an OD_600_ of ~ 3 achieved by strains SSK42 in 48 h. With respect to ethanol titer, *ptsG* deletion resulted in more than two times decrease in the final ethanol titer, i.e., maximum ethanol titers for SSK42 and SCD00 were 7.17 ± 0.28 g/L and 3.10 ± 0.29 g/L, respectively.

Overall, we observed a severe negative impact of *ptsG* deletion on total sugar consumption, thereby impacting growth and ethanol production. Nevertheless, a major positive impact of *ptsG* deletion apparent in the engineered strain was the glucose–xylose co-utilization, which was the primary objective of this study. However, co-utilization of sugars over a long period of time without significant product formation is of little use. Thus, we decided to improve the growth, total sugar consumption, and, hence, the ethanol production of the strain SCD00 via adaptive laboratory evolution. We observed that although consumption of both the sugars was affected by *ptsG* deletion, the glucose still remained the preferred sugar and was completely consumed before xylose. Hence, we decided to carry out the adaptation of SCD00 in xylose containing minimal medium to improve the xylose consumption.

### Adaptive laboratory evolution of the strain SCD00

The *ptsG* deleted engineered ethanologenic *E. coli* strain, i.e., SCD00, was subjected to adaptation in increasing concentration of xylose (Fig. 1), as xylose consumption rate was slower than glucose.

**Fig. 4:**
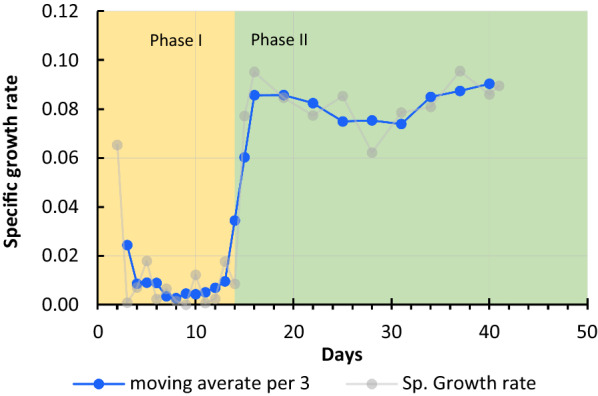
Change in the specific growth rate with passaging leading to strain SCD78. The moving average depicts the progressive change in the specific growth rate throughout the adaptation period. Actual data points are represented by light grey lines

As mentioned in "Materials and methods" section, the strain SCD00 was first evolved in CSTR at a very slow dilution rate, followed by passaging on agar plates. The strain was then passaged in Hungate tubes (containing 10 ml AM1 medium supplemented with 0.2% xylose), with all the biomass generated being added into the fresh medium every 24 h. Whole biomass passaging was continued for 14 days with xylose concentration increasing to 0.4% on 8th day of passaging. Due to total biomass transfer to fresh media, the extent of competition is higher even when the spent media is replenished with the fresh one every day. During the total biomass transfer phase of adaptation (phase I), the specific growth rate of the bacteria kept on declining to as low as 0.01 h^− 1^ and below (Fig. 4). Initial phase of evolution and total biomass transfer (phase I) would have helped to screen out the cells unsuitable for adaptation phase in growth conditions of choice. Bacterial cells which are robust enough to withstand the competition will still grow and take longer to enter stationary phase while less vigorous cells will enter stationary phase early or may simply die. Upon addition of fresh media, stationary phase cells take longer to start dividing again as compared to cells which are already in log phase or early stationary phase. This phase of rapid growth can be utilized to enrich comparatively robust cells in the given conditions for the adaptation experiment. We wanted to use this strategy to screen the healthy cells as an initial step of the adaptive laboratory evolution experiment.

The phase I of the ALE was primarily focused on screening in addition to adaptation, as discussed earlier. The limiting concentration of xylose and total biomass transfer would have provided the optimal conditions for the screening. During the phase I, culture growth kept on declining, and there was no point in completely arresting the culture growth. Therefore, the screening phase was maintained for seven days only (when growth rate reached the minimum) and xylose concentration was increased from 8th day onwards. Upon increasing the xylose concentration from 0.2 to 0.4% on 8th day of passaging, there was a slight impact on the specific growth rate (Fig. 4). On the 14th day and onwards, passaging was carried out with 0.1 OD_600_ being inoculated into the fresh medium (Phase II) every day for 41 days. A rapid increase in specific growth rate was observed on 15th day (Fig. 4). We reasoned that less inoculum being used for next passages resulted in the competition for resources becoming a bit lenient, hence the observed rapid increase in specific growth rate of the mutant bacteria.

The culture was adapted for a total of 48 days (including CSTR adaptation), during which the culture has undergone approximately 78 doublings as calculated using the following equation: 

1 $$\mu =\frac{\text{ln}\left({X}_{t}\right)-\text{l}\text{n}\left({X}_{t=0}\right)}{t},$$

2 $$Doubling \ time \left({T}_{g}\right)= \frac{0.693}{\upmu },$$

where µ is the specific growth rate, t is the time (h), X_t_ is dry cell weight (DCW) in g/L at time t. T_g_ represents the doubling time or generation time in Eq. (2).

The specific growth rate of the mutant strain remained close to 0.08 h^− 1^ throughout the phase II of the adaptation. We did the sampling on 41st day, which corresponded to approximately 78 generations and this strain was named SCD78. In a separate adaptation experiment, the strain SCD00 was also adapted on glucose followed by xylose for a total of 1200 generations to also improve the growth on glucose. However, it was observed that in this strain, named SCD1200, the carbon catabolite repression system seemed to have overwhelmed again and impacted the co-utilization of sugars (Additional file 1: Fig. S2), a phenotype worth investigating in future.

### Substrate co-utilization phenotype of adapted strain

Sugar consumption phenotype of the adapted strain was determined in a minimal medium containing a mixture of glucose and xylose (1.5 g/L each sugar) as carbon source. Experiment was performed in screw capped Hungate tubes to maintain micro-aerobic condition. Samples were removed from the tubes every three hours and analyzed for growth, sugars and metabolites as mentioned in methods section.

**Fig. 5:**
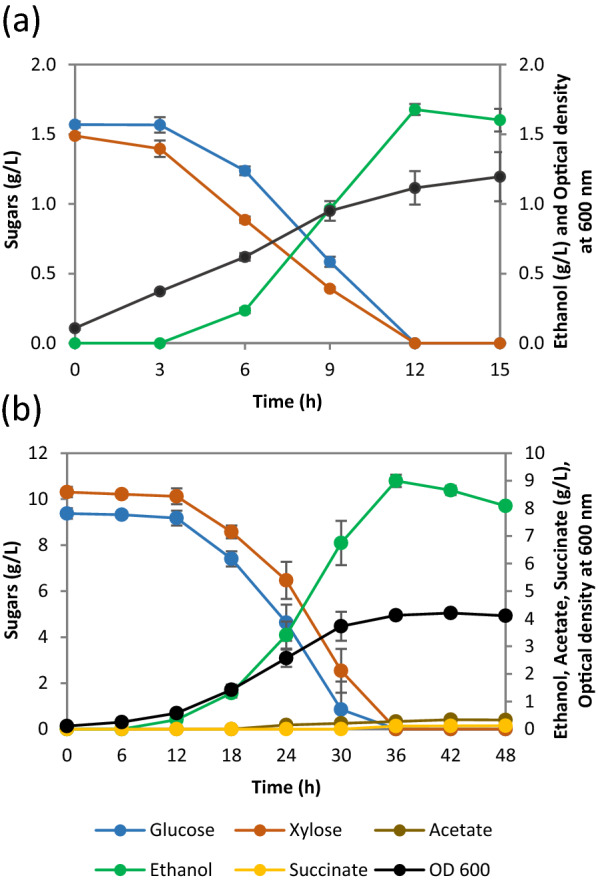
Fermentation profiles of strain SCD78. Fermentation profiles in Hungate tube (**a**) and bioreactor (**b**)

The evolved SCD78 strain doesn’t appear to favor any sugar over the other among glucose and xylose, instead, it consumed all the available sugar within 12 h by simultaneously consuming both the sugars- glucose and xylose at maximum consumption rate of 0.22 ± 0.00 and 0.17 ± 0.01 g/L/h respectively with a maximum ethanol production rate of 0.24 ± 0.00 g/L/h from 6th to 12th hour of fermentation (Fig. 5a). Strain SCD78 produced 23% higher ethanol titer in 12 h, i.e., 1.68 g/L, as compared to that achieved by strain SCD00 in 15 h (Fig. 2d).

Based on the phenotype in the Hungate tube experiment, the evolved strain SCD78 was selected for the bioreactor study under the control conditions. Strains were cultivated in a sugar mixture containing 10 g/L glucose and xylose each in AM1 minimal medium under controlled conditions as mentioned in methods section. The strain SCD78 generated by adaptive laboratory evolution of SCD00 showed a significantly improved growth profile compared to the control SCD00 strain (Figs. 3b, 5b; Table 3). It reached an OD_600_ of roughly 3 times that of the control strain, i.e., OD_600_ of 4.2, after 42 h.

Significant co-utilization was observed in both the strains at the reactor level (Figs. 3b, 5b), as opposed to the Hungate tube experiment, where SCD00 strain consumed glucose first with a minimal degree of co-utilization observed (Fig. 2d). Nevertheless, the strain SCD78 rapidly consumed both the sugars simultaneously within 36 h at a maximum sugar consumption rate of 0.63 ± 0.07 and 0.66 ± 0.02 g/L/h for glucose and xylose, respectively. The control strain (SCD00), on the other hand, consumed only about 5.4 ± 0.10 g/L of glucose and about 5.4 ± 0.16 g/L of xylose simultaneously in 48 h of fermentation. The maximum consumption rate achieved was only 0.23 ± 0.02 and 0.33 ± 0.08 g/L/h for glucose and xylose, respectively, indicating that strain SCD78 exhibited approximately 2.8 times higher glucose consumption rate and roughly 2 times higher xylose consumption rate.

**Table 3:** Fermentation parameters of SCD78 vs. SCD00 in bioreactor study

Strain	Sugar consumption rate (g/L/h)			Max. product titer (g/L)		Yield (g product/g total sugar consumed)		% Theoretical yield of ethanol	Max growth rate (g/L/h)	Max. volumetric ethanol productivity (g/L/h)
	Total sugar	Glucose	Xylose	Biomass	Ethanol	Biomass	Ethanol			
SCD00	0.26 ± 0.01	0.13 ± 0.00	0.13 ± 0.00	0.72 ± 0.00	3.10 ± 0.29	0.06 ± 0.00	0.29 ± 0.02	55.81 ± 3.92	0.05 ± 0.01 (12–18 h)	0.15 ± 0.01 (12–18 h)
SCD78	0.80 ± 0.03	0.44 ± 0.07	0.42 ± 0.01	2.10 ± 0.01	8.99 ± 0.22	0.11 ± 0.01	0.46 ± 0.02	84.35 ± 2.73	0.10 ± 0.01 (18–24 h)	0.56 ± 0.05 (24–30 h)

As expected, the strain SCD78 also performed better for ethanol production, with approximately 3 times higher ethanol titer observed after 36 h of fermentation. Strain SCD00 produced a maximum ethanol titer of 3.10 ± 0.29 g/L with maximum volumetric ethanol productivity of 0.15 ± 0.01 g/L/h achieved between 12 and 18 h of fermentation (Table 3). On the other hand, SCD78 produced 8.99 ± 0.22 g/L ethanol with maximum volumetric productivity 3.7 times that of the control strain (0.56 ± 0.05 g/L/h), achieved between 24 and 30 h of fermentation. The ethanol produced by the control strain (SCD00) and SCD78 corresponds to 55.81 ± 3.92% and 84.35 ± 2.73% of the theoretical limit. Apart from ethanol, small quantities of acetate and succinate were identified as products formed during fermentation. However, no succinate was detected for strain SCD00 (Additional file 1: Fig. S3).

### **Proteomic insights of engineered and adapted strains**

Deletion of *ptsG* led to co-utilization of sugars, and further adaptation on xylose not only improved the xylose consumption rate of the evolved strain but also improved the glucose consumption rate. To understand the reason for these cellular phenotypes, a whole cell proteome analysis was done for the strains SSK42, SCD00 and SCD78 to get an insight into the protein expression level. All the three strains were grown in a mixture of glucose and xylose (10 g/L each) in the bioreactor and samples were harvested in the early to mid-log phase of the growth (See methods section). The abundance values for each protein identified were normalized using quantile normalization algorithm and differential expression was compared using software named NormalyzerDE. Only the targets having log_2_ fold change of ≥ 2 with P-values ≤ 0.05 were considered for further analysis. All the differentially expressed proteins are mentioned in Additional file 1: Tables S1 and S2.

#### Comparison of protein profiles of SCD00 with respect to SSK42

##### Upregulated proteins

**Fig. 6:**
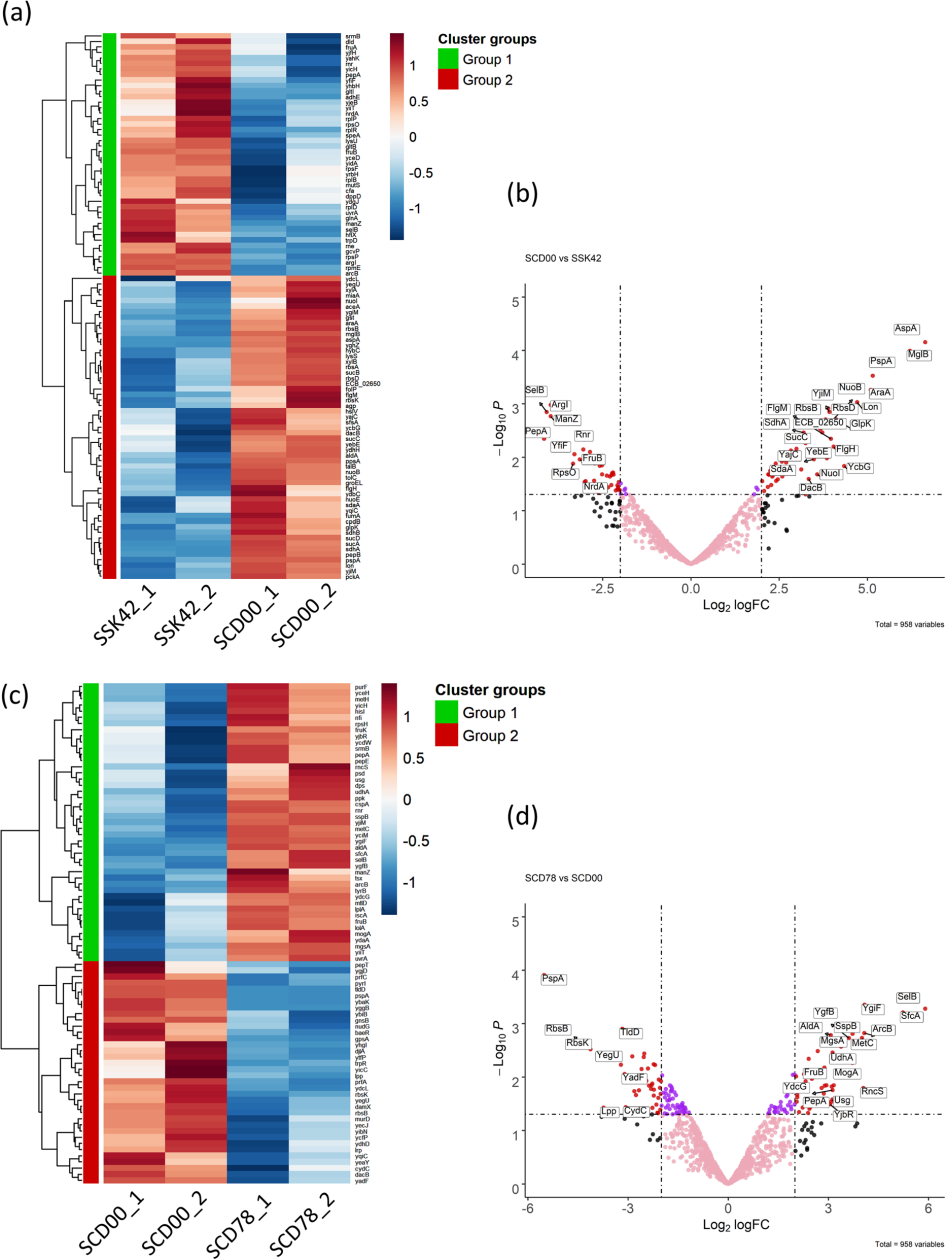
Analysis of the proteomics data for comparison of differentially expressed proteins. Heatmaps (**a**) and volcano plot (**b**) of all the significantly differentially expressed proteins in strain SCD00 in comparison to SSK42, and heatmap (**c**) and volcano plot (**d**) of all the identified proteins in the comparison group SCD78 vs. SCD00 is represented. Only the proteins with p-value less than 0.05 and log_2_ fold change ± 2 are represented. All the proteins with significant differential expression more than log_2_ fold change ± 2 are represented as red dots. Some of the important proteins have been highlighted with their names in the boxes on the volcano plot

**Fig. 7:**
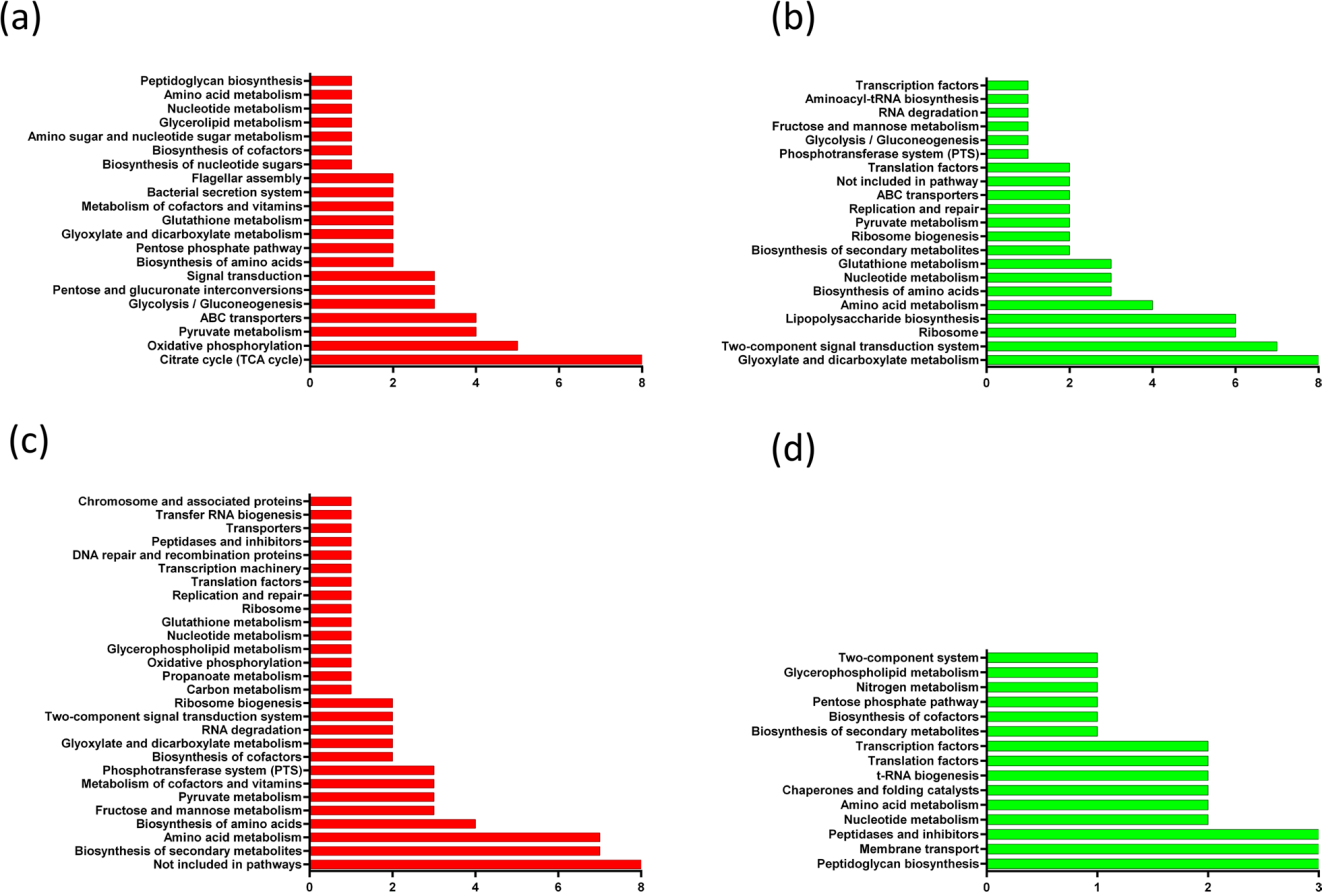
Pathway enrichment of differentially expressed proteins. Up-regulated (**a**) and down-regulated (**b**) proteins in strain SCD00 vs. SSK42, and upregulated (**c**) and downregulated (**d**) proteins in SCD78 vs. SCD00, respectively. In each of the plot, x-axis represents the number of proteins identified in each pathway category represented on y-axis

A total of 55 proteins were found to be overexpressed in SCD00 in comparison to SSK42 (Fig. 6a, b). Interestingly, the major pathways that got upregulated upon *ptsG* deletion are the energy yielding aerobic pathways. Citric acid cycle appears to be impacted the most since eight proteins belonging to citric acid cycle were significantly overexpressed (Fig. 7a). These eight proteins are FumA (Fumarate hydratase), SdhA (Succinate dehydrogenase subunit), SdhB (Succinate dehydrogenase; Fe-S subunit), SucD (Succinyl Co-A synthetase subunit α), SucC (Succinyl-CoA synthetase subunit β), SucB (Dihydrolipoamide acetyltransferase) and SucA (α-Ketoglutarate decarboxylase). These protein targets indicate up-regulation of TCA cycle flux from 2-oxoglutarate to malate which generates many important precursor metabolites and energy. The second most impacted pathway category is oxidative phosphorylation, followed by pyruvate metabolism and ABC transporters. Overexpression of NuoI (NADH dehydrogenase subunit I), NuoB (NADH dehydrogenase subunit B) and NuoE (NADH dehydrogenase subunit E) indicate the higher activity of NADH: quinone oxidoreductase system, which along with SdhA and SdhB overexpression indicates higher oxidoreductase activity in strain SCD00 compared to SSK42. YqiC (Ubiquinone biosynthesis accessory factor; UbiK), which is involved in biosynthesis of electron carrier ubiquinone pool [23], and HybC (Hydrogenase 2 large subunit), which is also the part of *E. coli* respiration system, were found to be overexpressed by approximately 2 log_2_ fold. This shows that there is significant re-arrangement in the respiration system-related proteins in strain SCD00 compared to SSK42.

Amino acid recycling proteins also were found to be upregulated. Aspartate ammonia-lyase (AspA) is overexpressed by more than 6 log_2_ fold. l-aspartate serves as high-quality nitrogen source under nitrogen limited conditions, and AspA is required to convert l-aspartate into fumarate and ammonia, thereafter, ammonia can serve as a source of precursor for l-glutamine, l-glutamate, nucleotides and amino sugars [24]. SdaA (l-serine deaminase I) overexpressed by almost 3 log_2_ fold catalyzes the conversion of the l-serine into pyruvate and ammonia.

The d-galactose/methyl-galactoside ABC transporter periplasmic binding protein (MglB) showed approximately 6 log_2_ fold overexpression, suggesting that it can act as a major glucose transporter inside the cell in a strain lacking the main glucose transporter gene *ptsG* [25, 26], hence an important target for increasing glucose utilization. Two gluconeogenic enzymes, PckA (Phosphoenolpyruvate carboxykinase) and PpsA (Phosphoenol pyruvate synthase) are also found to be significantly overexpressed. Both of these enzyme are reported to be important during diauxic shift of *E. coli* from glucose to acetate consumption [27]. TalB (Transaldolase B), an enzyme of pentose phosphate pathway (PPP), which along with transketolase, establishes a reversible link between PPP and glycolysis, is also found to be overexpressed by more than 2 log_2_ fold. The *ptsG* deletion also led to overexpression of proteins related to xylose consumption, i.e., XylA (Xylose isomerase) and XylB (Xylulokinase), which along with TalB overexpression indicates higher activity of PPP and supports the co-utilization phenotype of SCD00 for xylose and glucose. There is more than 3 log_2_ fold overexpression of proteins involved in the transport and metabolism of ribose sugar, such as – D-ribose periplasmic binding protein ([Locus_tag = ECB_02650), RbsA (Ribose ABC transporter ATP binding subunit), RbsB (D-ribose ABC transporter periplasmic binding protein) and RbsD (Ribose pyranase). GlpK (Glycerol kinase) was also overexpressed by 4 log_2_ fold in SCD00. There are reports that GlpK activity is inhibited in presence of un-phosphorylated EIIA^Glc^ but not the EIIA^Glc^-℗ [28, 29]. Therefore in the strain SCD00, which is supposed to have most of EIIA^Glc^ in phosphorylated form, i.e., EIIA^Glc^-℗, it may be possible that GlpK activity is not inhibited, and in addition, 4 log_2_ fold higher expression of GlpK may help in increasing the total carbon recovery by utilizing the glycerol moiety of phospholipids and triglycerides.

Lon (Lon protease) involved in protein homeostasis and quality control of proteins is found to be upregulated by more than 4 log_2_ fold. The protein YghZ (l-glyceraldehyde 3-phosphate reductase) is found to be overexpressed by more than 3 log_2_ fold. It has been hypothesized that YghZ might have role to play in detoxifying the toxic metabolite l-glyceraldehyde 3-phosphate and the methylglyoxal [30]. A broad specificity aldehyde dehydrogenase A (AldA; NAD-linked) is also found to be upregulated. It has been re-annotated to succinate semialdehyde dehydrogenase [31].

##### Downregulated proteins

A total of 44 proteins were significantly down-regulated, which include the members of glutamate transport and metabolism pathway - GltB (Glutamate synthase subunit) and GltI (Glutamate and aspartate transporter subunit), Fructose PTS transport system - FruA (Fused fructose specific PTS enzymes: IIB/IIC component), FruB (Fused fructose specific PTS: IIA/HPr component), ManZ (Mannose-specific enzyme IID component of PTS), Dld (D-lactate dehydrogenase; FAD-dependent) involved in lactate oxidation to pyruvate and ArgI (Ornitihine carbamoyltransferase I) involved in arginine biosynthesis (Figs. 6b, 7b). Ribosomal proteins like RpsP (30 S ribosomal protein S16), RpsO (30 S ribosomal protein S15), RpsF (30 S ribosomal protein S6), RplR (50 S ribosomal protein L18), RplD (50s ribosomal protein L4), RplP (50 S ribosomal protein L16), RpmE (50 S ribosomal protein L31), RplB (50 S ribosomal protein L2), YhbH (Predicted ribosome-associated, σ54 modulation protein) were found to be significantly down-regulated. This could be one of the reasons for diminished growth of SCD00 compared to SSK42. It could also be possible that the given amount of ribosomal machinery is enough for the cell, and it is in the interest of the cell to direct the extra energy for metabolic reshuffling in order to consume maximum sugar up to best of its capabilities. YidA, a promiscuous sugar phosphatase that is able to hydrolyze α-d-glucose-1-phosphate, is downregulated by more than 2 log_2_ fold [32]. Although another sugar phosphatase, Agp is also found to be overexpressed by more than 2 log_2_ fold (mentioned above), there are reports that it has 2 times higher K_*m*_ (lower affinity) to its substrate as compared to YidA [32]. AdhE (aldehyde/alcohol dehydrogenase) was also found to be down-regulated by 2.5 log_2_ fold. ArcB (DNA binding response regulator in two component regulatory system with ArcB and ArcA) is found downregulated. ArcB along with ArcA mediates repression of many aerobic enzymes under anaerobic conditions [33]. In fact, the observation in the previous subsection that many enzymes involved in TCA cycle and oxidative phosphorylation are upregulated in SCD00 could be due to downregulation of ArcB.

#### Comparison of protein profile of SCD78 with respect to SCD00

##### Upregulated proteins

Upon comparative proteomics analysis of strains SCD78 with respect to SCD00, a total of 45 proteins were found to be overexpressed (Fig. 6c, d). There are 10 proteins that were found to be significantly downregulated in SCD00 compared to SSK42 but are significantly overexpressed in strain SCD78 with respect to SCD00. These proteins include SelB, ArcB, FruB, PepA (Aminopeptidase A), YicH (Hypothetical protein), Rnr (RNase R), ManZ, SrmB (ATP dependent RNA helicase), YiiT (Stress induced protein) and UvrA (Excision nuclease A). These proteins may somehow help achieve strain SCD78 the growth advantage over SCD00 which the later lost to SSK42 due to deletion of *ptsG.* Two proteins, AldA (NAD^+^ dependent aldehyde dehydrogenase) and YjiM, were overexpressed in both the comparison groups signifying the positive correlation with *ptsG* deletion in strain SCD00 and adaptation in strain SCD78. Franchini et al. also found AldA to be upregulated in their study on bacterial adaptation in glucose limiting continuous cultures [34]. MaeA (Malate dehydrogenase, NAD^+^-requiring; SfcA), UdhA (Soluble pyridine nucleotide transhydrogenase), and MgsA (Methylglyoxal synthase) were found to be overexpressed by more than 3 log_2_ fold. The overexpression of MgsA and downregulation of GpsA appear to channel the DHAP towards methylglyoxal. Higher amount of methylglyoxal is an indication of accumulation of phosphorylated sugars inside the cell, which explains the higher total sugar consumption phenotype of SCD78. Adaptation of SCD00 to generate strain SCD78 appears to equilibrate the reducing power inside the cell, which is exemplified by overexpression of UdhA and Ppk (polyphosphate kinase). UdhA is primarily responsible for the reoxidation of NADPH in bacteria [35, 36].

Other overexpressed proteins which also appear to be responsible for a better growth profile of SCD78 include OpgD (Osmoregulated periplasmic glucan synthesis protein) involved in the osmoregulation [40], LolA (Outer-membrane lipoprotein carrier protein), FruK (1-Phosphofructokinase) which is essential for utilization of fructose as carbon source, PurF (Amidophosphoribosyl transferase) which play critical role in purine *de novo* biosynthesis, and LplA (lipoate-protein ligase A) required for proper functioning of many crucial enzymes involved in oxidative metabolism using exogenous lipoate. Enzymes involved in the synthesis of amino acids, such as histidine, methionine, leucine, tyrosine, and phenylalanine, were also found to be overexpressed in strain SCD78 as compared to SCD00. Downregulation of tryptophan operon repressor (TrpR) also indicate requirement for tryptophan biosynthesis.

##### Downregulated proteins

Proteins that were significantly overexpressed in SCD00 (as compared to SSK42) but were found to be downregulated in SCD78 (as compared to SCD00) include YqiC, PBP4 (Peptidoglycan DD endopeptidase; encoded by *dacB*) having both DD-endopeptidase and DD-carboxypeptidase activity and is involved in recycling and remodeling of gram-negative bacterial cell wall and proper separation of daughter cells [37–39], YdcL (Predicted lipoprotein), YegU (Predicted hydrolase), RbsB (d-ribose transporter subunit), RbsK (Ribokinase) and PspA (Regulatory protein for phage-shock-protein operon). RbsB and RbsK, belonging to RbsDACBK operon involved in ribose utilization, were found to be downregulated by more than 4 log_2_ fold. It appears that their upregulation due to *ptsG* deletion in SCD00 has been readjusted during the course of laboratory evolution in SCD78 since these proteins do not have direct role in either xylose or glucose utilization. Other downregulated proteins include PyrI (Aspartate carbamoyltransferase regulatory subunit), GpsA (NAD(P)H-dependent glycerol 3-phosphate dehydrogenase), Lrp (DNA-binding transcriptional dual regulator), YghL (Predicted gluconate transport associated protein), Can (Carbonic anhydrase, YadF) and few other proteins (Additional file 1: Table S2). The Can (β class carbonic anhydrase) interconverts CO_2_ and bicarbonate. Its expression is particularly required during growth in air, where partial pressure of CO_2_ is lower, and the requirement of bicarbonate is higher in the culture [40].

## Discussion

**Fig. 8:**
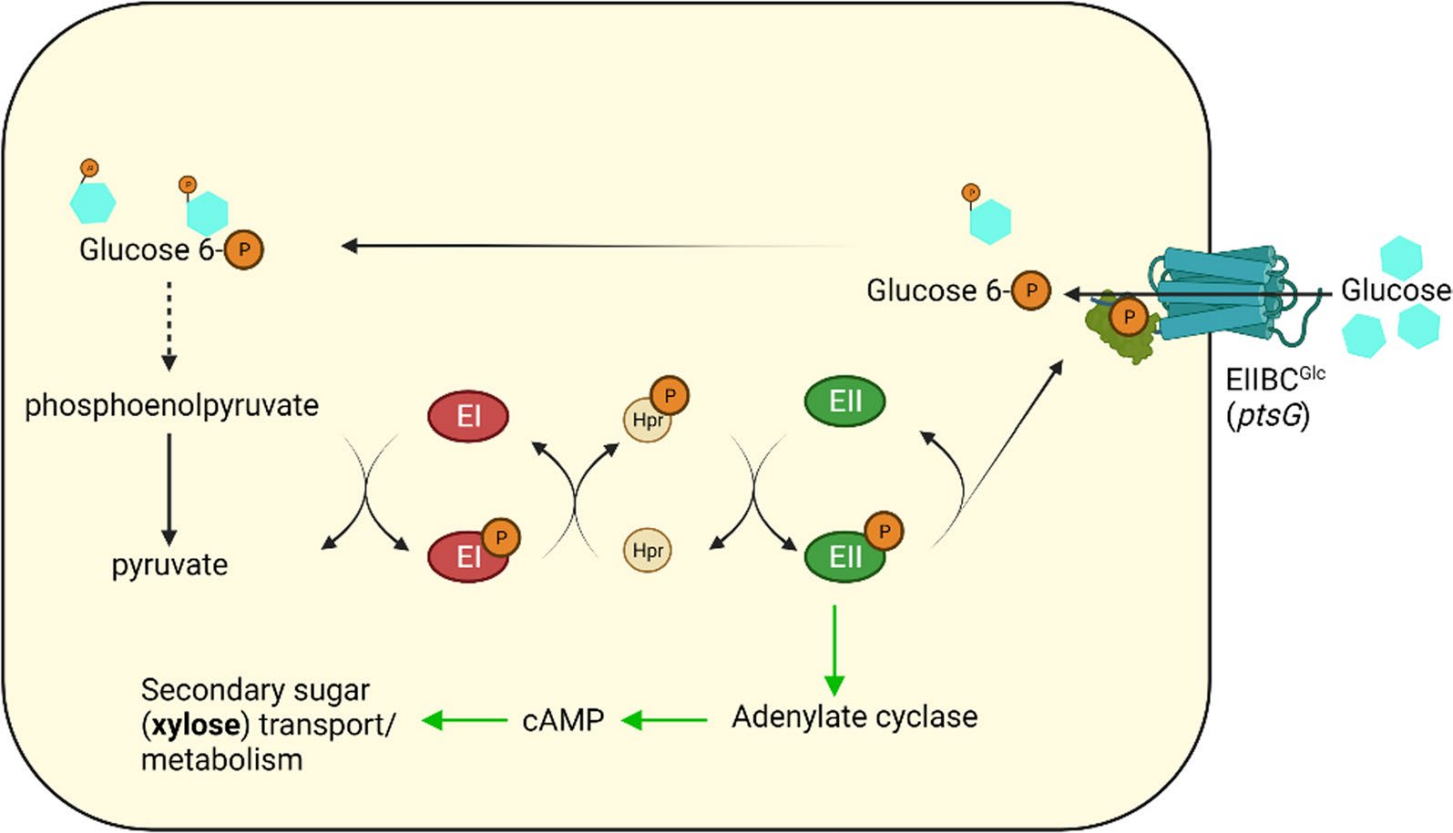
Schematic representation of glucose mediated catabolite repression through EIIA^Glc^ component of phosphotransferase system (PTS). The deletion of EIIBC^Glc^ component of PTS system may result in most of the EIIA^Glc^ remaining in phosphorylated form and, hence, may activate the xylose transport and metabolism (shown via green arrow). The EIIBC^Glc^ protein structure shown in the diagram is for representation only

Carbon catabolite repression (CCR) is an intricate regulation system in bacteria which manages the activity and expression of cellular enzymatic pools in a well-orchestrated manner for efficient sugar consumption and survival in various environmental conditions [41]. Glucose-specific PTS enzyme EIIBC (encoded by *ptsG*) is the membrane-associated subunit of the group translocation system of PEP-PTS (Phosphoenolpyruvate phosphotransferase system). EIIBC^Glc^ couples the vectorial translocation to the phosphorylation of the imported glucose molecules [42]. It is the phosphorylated form of EIIA^Glc^ that can act as an adenylate cyclase activator [43–45]. It also serves as activating signal for transporters and catabolic enzymes of secondary sugars. Therefore, we hypothesized that a deletion mutant of *ptsG* will have EIIA^Glc^ mostly in its phosphorylated form even in the presence of glucose in the culture medium since EIIBC^Glc^ is the primary acceptor of phosphate moiety from EIIA^Glc^ (Fig. 8). All of the EIIA^Glc^-℗ can then be used as an activating signal for transporters and catabolic enzymes of secondary sugars even in the presence of primary sugar glucose in the culture medium.

In this work, we used an ethanologenic strain SSK42 and made it CCR compromised by deleting *ptsG* encoding the glucose PTS transporter subunit (EIIBC^Glc^). The strain SCD00 (*ptsG* deleted SSK42 strain) displayed a significant co-utilization phenotype as compared to the parent strain in a sugar mixture containing 1% glucose and xylose each (Fig. 3b), but at lower sugar concentration, glucose is consumed first, followed by xylose with some degree of co-utilization still observed (Fig. 2d). The difference in the extent of co-utilization observed at higher and lower sugar concentrations implies that CCR may still be active to some extent even after the deletion of *ptsG*. Also, there could be cognate transporters that can potentially transport the glucose and xylose non-specifically at higher sugar concentration. Due to the higher degree of co-utilization observed in strain SCD00, we wished to improve its slower growth to further increase the total sugar consumption in a sugar mixture via the adaptive laboratory evolution strategy.

The strain SCD78, which has undergone approximately 78 generations in ALE on xylose, displays significant co-utilization at higher as well as lower sugar concentration as opposed to un-evolved strain SCD00, which only exhibits significant co-utilization at higher sugar concentrations, i.e., 1% glucose and xylose each. Although SCD78 has its glucose consumption delayed by 3 h compared to un-evolved strain SCD00 in Hungate tube experiments, its xylose consumption has increased by the same duration (Figs. 2d, 5a). Hence, it can consume total sugar faster than sequential sugar utilizers at low sugar concentrations. The strain SSK42 was earlier evolved on both glucose and xylose media for several generations and maintained on AM1 medium supplemented with xylose only [22] which could explain the rapid switch to xylose consumption after glucose and higher biomass generation. It is also worth noting that there is no significant change in the acetate production profiles of strain SCD78 as compared to the un-evolved control strain at the bioreactor level. However, there seems to be an increase in succinate production in SCD78 because no succinate was detected in SCD00 (Additional file 1: Fig. S3). It is interesting as *frdA*, which code for fumarate reductase responsible for conversion of fumarate to succinate under anaerobic condition, has been deleted in the SSK42 strain. It may indicate activation of alternative pathway for succinate production, perhaps due to the need for NAD^+^ regeneration. Succinate is also produced from succinic semialdehyde (SSA) via the γ-aminobutyric acid (GABA) shunt via glutamate. Under conditions of low oxygen, succinate can also accumulate due to reversed action of SDH. Considering that we observed overexpression of succinate semialdehyde dehydrogenase (NAD-linked AldA) in SCD78 in our proteomic studies, both of these pathways are possible for succinate production. Strain SCD78 produced 3 fold higher ethanol and biomass as compared to the control, with an ethanol yield of 84.35 ± 2.73% of the theoretical maximum limit as compared to the control (SCD00), which could only achieve an ethanol yield of approximately 55.8 ± 3.92% of the theoretical maximum limit.

**Fig. 9:**
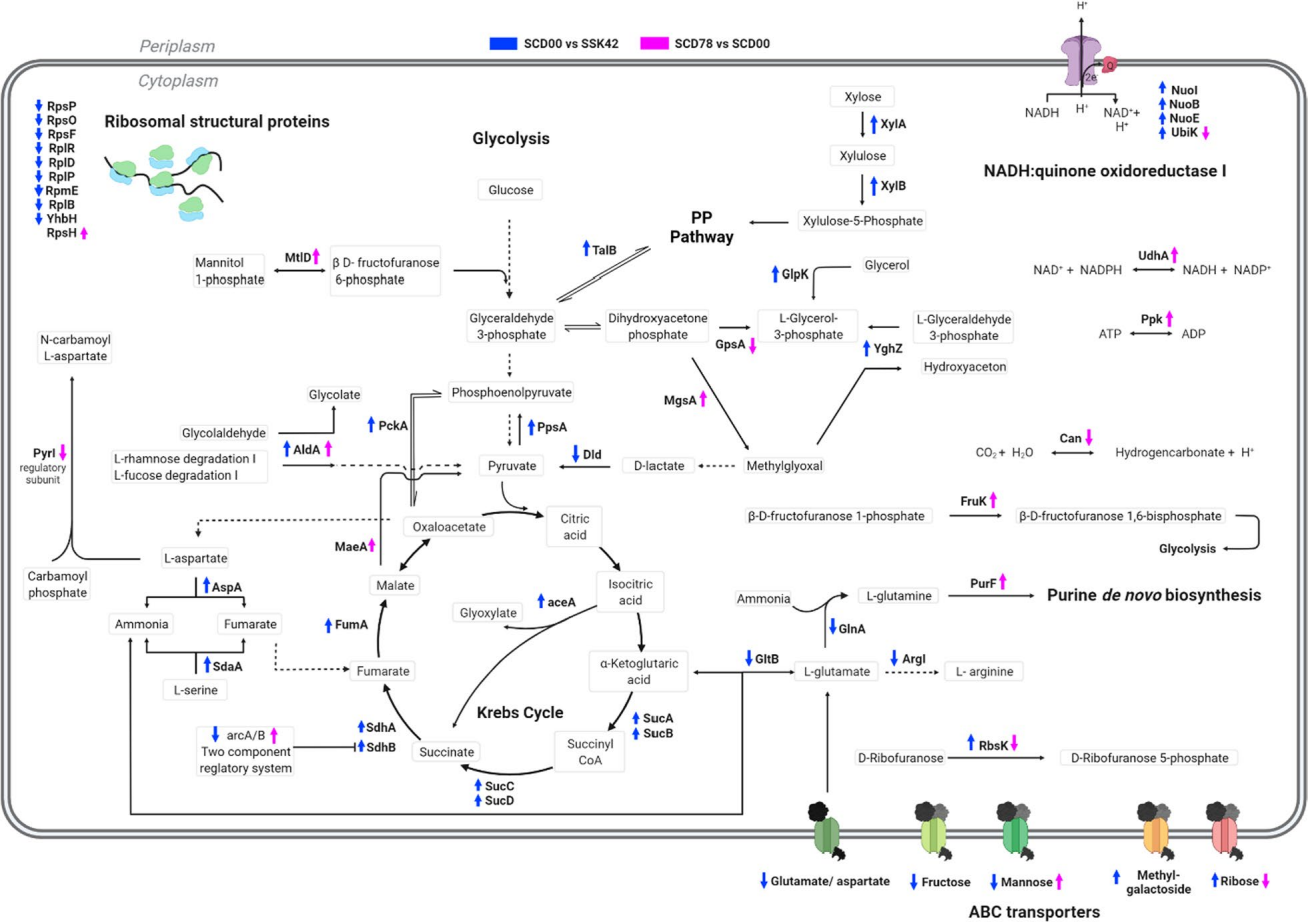
Schematic representation of metabolic pathway readjustment in strain SCD00 and SCD78. The pathway map represents the metabolic reactions which are differentially regulated at enzymatic level. There are two comparison groups depicted on the pathway map—SCD00 vs. SSK42 and SCD78 vs. SCD00

The whole-cell proteome comparison indicates that *ptsG* has led to higher flux through the TCA cycle and oxidative phosphorylation in strain SCD00 (Fig. 9). This could be due to the downregulation of the global regulator ArcB, which suppresses the aerobic enzymes expression under oxygen limiting conditions. Overexpression of xylose consumption proteins and transaldolase B (TalB) in SCD00 strain also indicates higher PP pathway flux (Fig. 9). These observations along with recycling of aspartate and serine amino acids, creates an impression of significant redistribution of metabolite flux to accommodate xylose sugars along with the glucose inside the cell. For generating the energy to sustain the cellular machinery, there is an increased respiration capacity, particularly NADH:quinone oxidoreductase I system. The downregulation of ABC transporters such as glutamate/aspartate, fructose, and mannose ABC transporter could be to save the energy from getting wasted on transporters whose substrate is not even supplied in the culture medium. In absence of a working glucose-PTS system, overexpressed (6 log_2_ fold) methyl-galactoside ABC transporter could be responsible for glucose import.

After adaptation on xylose, the observed proteomics changes are not as drastic as for strain SCD00. It could be possible that the final phenotype is not only the result of differential protein expression but some genomic changes might be involved. Some minor but crucial changes which imparted the efficient sugar consumption and better growth profile for strain SCD78 are but not limited to—proper balancing the reducing power inside the cell by interconverting NADH and NADPH, also ATP and ADP, increase in purine de novo biosynthesis, increase in amino acid biosynthesis, overexpression of mannose ABC transporter and further reshaping of TCA cycle flux by overexpressed MaeA (Malate dehydrogenase, NAD^+^ requiring) (Fig. 9). Upregulation of ArcA/B regulation system along with better management of reducing power could be the factor responsible for higher sugar consumption, higher ethanol yield and better growth profile of strain SCD78. UdhA has been implicated in restoring the redox balance in the cell by catalyzing the electron transfer from NADPH to NADH [36]. The over-expression of UdhA (approximately 3.1 log_2_ fold) in strain SCD78 compared to SCD00 could be the consequence of the higher flux through the pentose phosphate pathway (PPP) for the need of redox balance being met, i.e., converting excess NADPH to NADH. Higher PPP flux explains the glucose–xylose co-utilization and overall higher sugar consumption rate of strain SCD78 compared to SCD00. Higher sugar consumption requires higher sugar transport and a higher metabolism rate. If the downstream metabolism is slower than required, the phosphorylated sugars may build up inside the cell. Methylglyoxal production is generally considered as a strategy to avoid stress due to the accumulation of phosphorylated sugars inside the cell. Methylglyoxal is extremely toxic to cells, but its controlled expression is viewed as a defense mechanism against the accumulation of phosphorylated sugars inside the cell, which is also deleterious to cell survival [46]. The formation of methylglyoxal at levels non-toxic to the cell channels the extra phosphorylated sugars, which would otherwise cause metabolic stress to the cell [46–48]. Overexpression of MgsA in SCD78 signifies the presence of a higher amount of phosphorylated sugars as compared to SCD00 (Fig. 9), which to an extent explains the higher sugar consumption rate of strains SCD78. Kim et al. have demonstrated that overexpression of *rbsDACBK* operon (containing genes for the transport and initial-step metabolism of d-ribose) on multicopy plasmids results in cell death after ribose addition to the media due to methylglyoxal production at lethal levels [49]. In line with that observation, we found approximately 4 log_2_ fold downregulation of RbsK and RbsB, although MgsA is still overexpressed by more than three log_2_ fold. It is to be noted that we have not added any external ribose to the culture media. Whether methylglyoxal production is helpful or not is yet to be determined in our case. However, Ingram et al. have already shown that the deletion of MgsA imparts the glucose–xylose co-utilization phenotype in a recombinant *E. coli* strain expressing *Zymomonas mobilis* ethanol pathway (*pdc*, *adhA*, and *adhB*) [50]. The deletion of *mgsA* can be explored to further improve the ethanol production in strain SCD78.

In conclusion, we found that deletion of *ptsG* gene can severely impact glucose utilization but pave the way for xylose utilization. The impact of deletion was much more pronounced on the wild type strain *E. coli* B as compared to the engineered and evolved ethanologenic strain SSK42. Adaptive evolution on xylose as the only sugar, was found to be a helpful strategy to develop a glucose–xylose co-utilizing strain in a minimal media. Finally, the proteomic studies of the engineered and evolved strains provided insights into the phenotypic changes that led to the development of a successful co-utilizing strain.

## Materials and methods

### Culture media

All the growth and phenotype determination studies were done in AM1 minimal medium supplemented with glucose and/or xylose, as mentioned in the result section. Composition of AM1 minimal media is as follows: (NH_4_)_2_HPO_4_ − 19.92 mM, NH_4_H_2_PO_4_ − 7.56 mM, KCl − 2.0 mM, MgSO_4_.7H_2_O − 1.5 mM, betaine HCl − 1.00 mM, FeCl_3_.6H_2_O − 8.88 µM, CoCl_2_.6H_2_O − 1.26 µM, CuCl_2_.2H_2_O − 0.88 µM, ZnCl_2_ − 2.20 µM, Na_2_MoO_4_.2H_2_O − 1.24 µM, H_3_BO_3_ − 1.21 µM, MnCl_2_.4H_2_O − 2.50 µM and CaCl_2_.2H_2_O − 1.36 µM [51]. For maintaining the cultures on solid media plates, AM1 agar plates containing 20 g/L xylose as carbon source were used. All the strains used in this study were maintained on AM1 agar plates except during phage mediated transduction and regeneration after transformation. In those cases, the associated protocols were followed or LB medium plates with appropriate antibiotics were used. All chemicals used were of analytical grade.

### Bacterial strains

Earlier, our group reported the development of an ethanologenic strain named SSK42 through Adaptive Laboratory Evolution (ALE) [22]. This strain is a derivative of *E. coli* B strain. The strain SSK42 has *ldhA, frdA* and *pflB* genes deleted, and the promoter of *pdh* operon is replaced with that of *gapA* gene for constitutive expression in microaerobic conditions. In this study, we further deleted *ptsG* gene from SSK42 by phage-mediated transduction method using the single gene Keio knockout strain (JW1087) from CGSC (Yale University, USA) as donor strain and confirmed the knockout on agarose gel electrophoresis after PCR amplification. The strain SCD00 was then adapted (as mentioned elsewhere) to generate strain SCD78, where numbers following the letters denote the number of doublings the strains have undergone to reach the current status. All the strains used in this study are mentioned in Table 1.

### Hungate tube culture

Screwcap Hungate tubes of capacity 18 mL with the working volume of the culture as 10 mL were used in the study. After inoculation, the tubes were tightly capped and incubated at 37 °C on a test tube rotator rotating the tubes by 360 °C vertically on its transverse axis at 25–30 RPM (rotations per minute).

### Adaptive laboratory evolution (ALE) experiment

All adaptation related work was done in AM1 minimal medium (solid as well as liquid). Strain SCD00 was evolved using a combination of adaptation steps, which are as follows.

Strain was evolved in Continuous Stirred Tank Reactor (CSTR) at a very slow dilution rate, limited by sugar concentration. The Applikon 500 mL capacity bioreactor was used for the purpose with working volume of 300 mL. The air is supplied only in the headspace at 0.03–0.04 LPM (liters per minute) to keep micro-aerobic conditions in the bioreactor. Agitation was done using Rushton type impellers at 300 rpm. The pH did not vary much during the fermentation and it remained near the initial value of pH 6.8, because fresh medium was continuously added to the reactor. Xylose was used as sole carbon at 1.0 g/L in the feed. The CSTR was kept running for 7 days at a dilution rate of 0.01 h^− 1^. Sample was obtained from the CSTR after 7 days and stored in the form of glycerol stock at − 80 °C, and the strain was named SCD00_ada. Later, the strain SCD00_ada was passaged on AM1 agar plates (20 g/L xylose) for 10 days and then adapted in Hungate tubes containing AM1 medium supplemented with 2 g/L of xylose. For first 14 days of adaptation in Hungate tubes, whole biomass from the previous day was added to fresh media each day (xylose concentration increased to 4 g/L on 8th day onwards). For next 27 days, only the culture volume equivalent to an OD_600_ of 0.1 from the previous day culture was added to fresh media every day. After neglecting the generations passed on AM1 agar plates, the whole duration of adaptation starting with CSTR resulted in approximately 78 generations of the culture. Hence, the strain obtained was named SCD78.

### Analytical assays

Sugars (glucose and xylose) and metabolite – acetic acid, succinic acid, lactic acid, and ethanol concentrations were determined using HPLC (Shimadzu) connected with Aminex HPX 87 H (300 × 7.8 mm) column and RI detector. The column temperature was maintained at 40 °C and 4 mM H_2_SO_4_ was used as mobile phase at 0.3 mL/min flow rate. Samples were drawn at required time points from bioreactor and immediately filtered through 0.2 μm syringe filters. If required, the appropriate dilutions of filtered samples were prepared in 4 mM H_2_SO_4_ and 10 µL were injected into HPLC. The quantification of each metabolite was done using reference standards of concentration 1 g/L obtained from Absolute Standards, USA. Culture growth was determined by measuring the optical density at 600 nm (OD_600_) using spectrophotometer (GE Healthcare).

### Bioreactor studies

Bioreactor cultivation studies were performed in multivessel fermenter assembly (Multifors, from Infors, USA). Each vessel has 500 mL volumetric capacity. For cultivation, the culture was first passaged on AM1 medium agar plates supplemented with 20 g/L xylose as sole carbon source for at least three passages. It was followed by primary culture in Hungate tubes with 10 mL AM1 medium supplemented with 20 g/L xylose, incubated at 37 °C at 25 RPM for 12–18 h. This tube culture was then used to inoculate the primary fermenter by adding all the tube culture to the fermenter. Primary fermenter was done in 10 g/L of xylose as sole carbon source in 300 mL of culture volume. Primary fermenter was allowed to grow overnight or till the culture reached an OD_600_ of approximately 2.0. Then, the final secondary reactor was inoculated with an appropriate culture volume from primary fermenter after washing the inoculum with fresh media by pelleting at 4500 RPM for 5 min in a tabletop centrifuge at room temperature. Starting OD_600_ was kept at 0.1 in the final reactor. This reactor had a mixture of glucose and xylose at 10 g/L each as carbon sources. Kanamycin was used wherever necessary at 50 mg/L concentration. Fermenter was maintained at 37 ˚C prior to the inoculation. Primary as well as secondary fermenter cultivation was done at 37 °C at 300 impeller rpm. Air was only supplied in the headspace at 0.03–0.04 LPM (liters per minute). Sampling was done at required time points using *Supersafe* autosamplers supplied with one way valve to prevent accidental contamination of the culture. The samples were immediately filtered through 0.2 μm syringe filters and analyzed through HPLC for metabolite and sugars or stored at − 20 °C. Also, approximately 1 mL of sample was used to determine the growth by checking the optical density at 600 nm.

### Whole cell proteomics

Strains SSK42, SCD00 and SCD78 were grown in bioreactor in presence of sugar mixture comprising of glucose and xylose (10 g/L each sugar) with same strategy as mentioned in the above section. Cultures were grown until early to mid-log phase of growth (OD_600_ of 0.3–0.8) which was achieved in 6 to 11 h. Culture was immediately centrifuged and pellet collected at 4 °C. The pellet was washed twice with cold MQ water and re-suspended in sonication buffer. The sonication buffer included Triton X-100 (1% v/v), Sodium chloride (150 mM), Tris-HCl pH-7.4 (50 mM), EDTA (1 mM), PMSF (1 mM), Lysozyme (1 mg/mL), 1,4-Dithiothreitol (1 mM) and *Phos-stop* tablets (anti phosphatases) from *Pierce.* Cell suspension equivalent to an OD_600_ of 75–100 was taken in a Pyrex tube and volume was increased to 5 mL (final OD_600_ – 15 to 20) with cold sonication buffer. Culture was maintained on ice all time. Sonication was performed for 3 min with 3 s ON/5 s OFF cycle at 20% amplitude. After effective lysis, lysate was centrifuged at 12,000*g* for 10 min at 4 °C in a table top centrifuge, and supernatant was collected in Eppendorf tubes. Protein content was determined using BCA kit (G-Biosciences) following the manufacturer’s instructions. A total 50 µg protein was taken per reaction to proceed further for reduction, alkylation, and digestion. Appropriate volume of protein sample (lysate supernatant) was diluted with urea (10 M in 50 mM ammonium bicarbonate solution) to a final volume of 150 µL. DTT was added to a final concentration of 10 mM and gently mixed and incubated at 56 °C for 45 min. Then, iodoacetamide (200 mM stock in 50 mM ammonium bicarbonate) was added to 50 mM final concentration, gently mixed, and incubated at room temperature in the dark for 45 min. DTT (30 mM final concentration) was added again and incubated at room temperature in the dark for 45 min to consume any unreacted iodoacetamide. Calcium chloride (1 mM in 50 mM ammonium bicarbonate solution) was added to the reaction to reduce the urea concentration to < 0.5 M. Protein solution was then subjected to trypsin digestion. Trypsin from *Pierce* was used for the purpose in a 1:50 (w/w trypsin: protein) ratio as suggested by the manufacturer, and reaction content was incubated at 37 °C overnight. Two drops of concentrated formic acid were added to lower the reaction pH and stop the trypsin reaction. C18 columns from *Pierce* were used for sample cleanup before being analyzed on mass spectrometer (Orbitrap Fusion™ Lumos™ from ThermoFisher Scientific).

## Supplementary information

**Additional file 1: Fig. S1: **Sugar consumption, ethanol production and growth profile. Strain SSK42 (A) and SCD00 (B). Growth profile is represented by OD600. **Fig. S2**: Sugar consumption, ethanol production and growth profile of strain SCD1200 that has been evolved on xylose and glucose in sequence. Sugars concentrations are plotted on left y-axis, while ethanol and optical density are plotted on the right y-axis. Optical density represents the growth profile. Data represents the average of two biological replicates. **Fig. S3**: Metabolite production profiles of strains SCD00 and SCD78. Solid lines represent the strain SCD78 while dotted lines represent the SCD00 strain. **Table S1**: Differentially expressed protein in strain SCD00 vs. SSK42. Only the protein with P Value lesser than 0.05 and log_2_ fold change of more than ± 2 are represented. **Table S2**: Differentially expressed protein in strain SCD78 vs. SCD00. Only the protein with P Value lesser than 0.05 and log_2_ fold change of more than ± 2 are represented.

## Data Availability

The mass spectrometry proteomics data has been deposited to the ProteomeXchange Consortium via the PRIDE [52] partner repository with the dataset identifier PXD033577 and 10.6019/PXD033577.

## References

[1] Jeswani HK, Chilvers A, Azapagic A (2020). Environmental sustainability of biofuels: a review. Proc R Soc A..

[2] Hassan SS, Williams GA, Jaiswal AK (2018). Emerging technologies for the pretreatment of lignocellulosic biomass. Bioresour Technol.

[3] Kumar AK, Sharma S (2017). Recent updates on different methods of pretreatment of lignocellulosic feedstocks: a review. Bioresour Bioprocess..

[4] Menon V, Rao M (2012). Trends in bioconversion of lignocellulose: biofuels, platform chemicals & biorefinery concept. Prog Energy Combust Sci.

[5] Esquivel-Hernández DA, García-Pérez JS, López-Pacheco IY, Iqbal HMN, Parra-Saldívar R (2022). Resource recovery of lignocellulosic biomass waste into lactic acid - Trends to sustain cleaner production. J Environ Manage..

[6] Pratto B, Chandgude V, de Sousa R, Cruz AJG, Bankar S (2020). Biobutanol production from sugarcane straw: defining optimal biomass loading for improved ABE fermentation. Ind Crops Prod.

[7] Kawaguchi H, Takada K, Elkasaby T, Pangestu R, Toyoshima M, Kahar P (2022). Recent advances in lignocellulosic biomass white biotechnology for bioplastics. Bioresour Technol..

[8] Mazzoli R (2020). Metabolic engineering strategies for consolidated production of lactic acid from lignocellulosic biomass. Biotechnol Appl Biochem.

[9] Thygesen A, Tsapekos P, Alvarado-Morales M, Angelidaki I (2021). Valorization of municipal organic waste into purified lactic acid. Bioresour Technol..

[10] Narisetty V, Parhi P, Mohan B, Hakkim Hazeena S, Naresh Kumar A, Gullón B (2022). Valorization of renewable resources to functional oligosaccharides: recent trends and future prospective. Bioresour Technol.

[11] Sànchez Nogué V, Karhumaa K (2015). Xylose fermentation as a challenge for commercialization of lignocellulosic fuels and chemicals. Biotechnol lett.

[12] Alterthum F, Ingram LO (1989). Efficient ethanol production from glucose, lactose, and xylose by recombinant *Escherichia coli*. Appl Environ Microbiol.

[13] Kim JH, Block DE, Mills DA (2010). Simultaneous consumption of pentose and hexose sugars: an optimal microbial phenotype for efficient fermentation of lignocellulosic biomass. Appl Microbiol Biotechnol..

[14] Pratish G, Patrick H, Andrew E, Martin VJJ, Mahadevan R (2013). Novel approach to engineer strains for simultaneous sugar utilization. Metab Eng..

[15] Apel AR, Ouellet M, Szmidt-Middleton H, Keasling JD, Mukhopadhyay A (2016). Evolved hexose transporter enhances xylose uptake and glucose/xylose co-utilization in *Saccharomyces cerevisiae*. Sci Rep..

[16] Wang M, Yu C, Zhao H (2016). Directed evolution of xylose specific transporters to facilitate glucose–xylose co-utilization. Biotechnol Bioeng..

[17] Nichols NN, Dien BS, Bothast RJ (2001). Use of catabolite repression mutants for fermentation of sugar mixtures to ethanol. Appl Microbiol Biotechnol.

[18] Lu H, Zhao X, Wang Y, Ding X, Wang J, Garza E (2016). Enhancement of D-lactic acid production from a mixed glucose and xylose substrate by the *Escherichia coli* strain JH15 devoid of the glucose effect. BMC Biotechnol..

[19] Li F-F, Zhao Y, Li B-Z, Qiao J-J, Zhao G-R (2016). Engineering *Escherichia coli* for production of 4-hydroxymandelic acid using glucose–xylose mixture. Microb Cell Fact..

[20] Nichols NN, Dien BS, Bothast RJ (2001). Use of catabolite repression mutants for fermentation of sugar mixtures to ethanol. Appl Microbiol Biotechnol.

[21] Munjal N, Mattam AJ, Pramanik D, Srivastava PS, Yazdani SS (2012). Modulation of endogenous pathways enhances bioethanol yield and productivity in *Escherichia coli*. Microb Cell Fact.

[22] Jilani SB, Venigalla SSK, Mattam AJ, Dev C, Yazdani SS (2017). Improvement in ethanol productivity of engineered *E. coli* strain SSY13 in defined medium via adaptive evolution. J Ind Microbiol Biotechnol.

[23] Loiseau L, Fyfe C, Aussel L, Chehade MH, Hernández SB, Faivre B (2017). The UbiK protein is an accessory factor necessary for bacterial ubiquinone (UQ) biosynthesis and forms a complex with the UQ biogenesis factor UbiJ. J Biol Chem..

[24] Schubert C, Zedler S, Strecker A, Unden G (2021). L-Aspartate as a high-quality nitrogen source in *Escherichia coli*: regulation of L-aspartase by the nitrogen regulatory system and interaction of L-aspartase with GlnB. Mol Microbiol..

[25] Gosset G (2005). Improvement of *Escherichia coli* production strains by modification of the phosphoenolpyruvate:sugar phosphotransferase system. Microb Cell Fact.

[26] Wick LM, Quadroni M, Egli T (2001). Short- and long-term changes in proteome composition and kinetic properties in a culture of *Escherichia coli* during transition from glucose-excess to glucose-limited growth conditions in continuous culture and vice versa. Environ Microbiol.

[27] Kao KC, Tran LM, Liao JC (2005). A global regulatory role of gluconeogenic genes in *Escherichia coli* revealed by transcriptome network analysis. J Biol Chem..

[28] Novotny MJ, Frederickson WL, Waygood EB, Saier MH (1985). Allosteric regulation of glycerol kinase by enzyme IIIglc of the phosphotransferase system in *Escherichia coli* and *Salmonella typhimurium*. J Bacteriol.

[29] De Boer M, Broekhuizen CP, Postma PW (1986). Regulation of glycerol kinase by enzyme IIIGlc of the phosphoenolpyruvate:carbohydrate phosphotransferase system. J Bacteriol.

[30] Desai KK, Miller BG (2008). A metabolic bypass of the triosephosphate isomerase reaction. Biochemistry..

[31] Reed JL, Vo TD, Schilling CH, Palsson BO (2003). An expanded genome-scale model of *Escherichia coli* K-12 (iJR904 GSM/GPR). Genome Biol.

[32] Kuznetsova E, Proudfoot M, Gonzalez CF, Brown G, Omelchenko MV, Borozan I (2006). Genome-wide analysis of substrate specificities of the *Escherichia coli* haloacid dehalogenase-like phosphatase family. J Biol Chem.

[33] Iuchi S, Matsuda Z, Fujiwara T, Lin ECC (1990). The arcB gene of *Escherichia coli* encodes a sensor-regulator protein for anaerobic repression of the arc modulon. Mol Microbiol.

[34] Franchini AG, Egli T (2006). Global gene expression in *Escherichia coli* K-12 during short-term and long-term adaptation to glucose-limited continuous culture conditions. Microbiology.

[35] Sauer U, Canonaco F, Heri S, Perrenoud A, Fischer E (2004). The soluble and membrane-bound transhydrogenases UdhA and PntAB have divergent functions in NADPH metabolism of *Escherichia coli*. J Biol Chem.

[36] Canonaco F, Hess TA, Heri S, Wang T, Szyperski T, Sauer U (2001). Metabolic flux response to phosphoglucose isomerase knock-out in *Escherichia coli *and impact of overexpression of the soluble transhydrogenase UdhA.. FEMS Microbiol Lett.

[37] Korat B, Mottl H, Keck W (1991). Penicillin-binding protein 4 of *Escherichia coli*: molecular cloning of the dacB gene, controlled overexpression, and alterations in murein composition. Mol Microbiol.

[38] Dik DA, Fisher JF, Mobashery S (2018). Cell-wall recycling of the gram-negative bacteria and the nexus to antibiotic resistance. Chem Rev..

[39] Priyadarshini R, Popham DL, Young KD (2006). Daughter cell separation by penicillin-binding proteins and peptidoglycan amidases in *Escherichia coli*. J Bacteriol.

[40] Merlin C, Masters M, McAteer S, Coulson A (2003). Why is carbonic anhydrase essential to *Escherichia coli*?. J Bacteriol..

[41] Deutscher J, Francke C, Postma PW (2006). How phosphotransferase system-related protein phosphorylation regulates carbohydrate metabolism in bacteria. Microbiol Mol Biol Rev..

[42] Meins$ M, Jenos P, Miillera D, Richter, Rosenbusch$ WJ, Erni JP (1993). Cysteine phosphorylation of the glucose transporter of *Escherichia coli*. J Biol Chem.

[43] Reddy P, Kamireddi M (1998). Modulation of *Escherichia coli* adenylyl cyclase activity by catalytic-site mutants of protein IIA(Glc) of the phosphoenolpyruvate:sugar phosphotransferase system. J Bacteriol.

[44] Takahashi H, Inada T, Postma P, Aiba H (1998). CRP down-regulates adenylate cyclase activity by reducing the level of phosphorylated IIA(Glc), the glucose-specific phosphotransferase protein, in *Escherichia coli*. Mol Gen Genet.

[45] Park YH, Lee BR, Seok YJ, Peterkofsky A (2006). In vitro reconstitution of catabolite repression in *Escherichia coli*. J Biol Chem.

[46] Tötemeyer S, Booth NA, Nichols WW, Dunbar B, Booth IR (1998). From famine to feast: the role of methylglyoxal production in *Escherichia coli*. Mol Microbiol..

[47] Boulanger EF, Sabag-Daigle A, Thirugnanasambantham P, Gopalan V, Ahmer BMM (2021). Sugar-phosphate toxicities. Microbiol Mol Biol Rev..

[48] Fraenkel DG (1968). The accumulation of glucose 6-phosphate from glucose and its effect in an *Escherichia coli* mutant lacking phosphoglucose isomerase and glucose 6-phosphate dehydrogenase. J Biol Chem.

[49] Kim I, Kim E, Yoo S, Shin D, Min B, Song J (2004). Ribose utilization with an excess of mutarotase causes cell death due to accumulation of methylglyoxal. J Bacteriol..

[50] Yomano LP, York SW, Shanmugam KT, Ingram LO (2009). Deletion of methylglyoxal synthase gene (mgsA) increased sugar co-metabolism in ethanol-producing *Escherichia coli*. Biotechnol Lett..

[51] Martinez A, Grabar TB, Shanmugam KT, Yomano LP, York SW, Ingram LO (2007). Low salt medium for lactate and ethanol production by recombinant *Escherichia coli* B. Biotechnol Lett.

[52] Perez-Riverol Y, Bai J, Bandla C, García-Seisdedos D, Hewapathirana S, Kamatchinathan S (2022). The PRIDE database resources in 2022: a hub for mass spectrometry-based proteomics evidences. Nucleic Acids Res.

