# Supplementary material for: Adaptation on xylose improves glucose–xylose co-utilization and ethanol production in a carbon catabolite repression (CCR) compromised ethanologenic strain
Source: Microb Cell Fact. 2022 Aug 6;21:154. doi: 10.1186/s12934-022-01879-1 (PMC9356451; doi:10.1186/s12934-022-01879-1)
Supplement: Supplementary file 1 — Additional file 1: Fig. S1: Sugar consumption, ethanol production and growth profile. Strain SSK42 (A) and SCD00 (B). Growth profile is represented by OD600. Fig. S2: Sugar consumption, ethanol production and growth profile of strain SCD1200 that has been evolved on xylose and glucose in sequence. Sugars concentrations are plotted on left y-axis, while ethanol and optical density are plotted on the right y-axis. Optical density represents the growth profile. Data represents the average of two biological replicates. Fig. S3: Metabolite production profiles of strains SCD00 and SCD78. Solid lines represent the strain SCD78 while dotted lines represent the SCD00 strain. Table S1: Differentially expressed protein in strain SCD00 vs. SSK42. Only the protein with P Value lesser than 0.05 and log2 fold change of more than ± 2 are represented. Table S2: Differentially expressed protein in strain SCD78 vs. SCD00. Only the protein with P Value lesser than 0.05 and log2 fold change of more than ± 2 are represented. [file 12934_2022_1879_MOESM1_ESM.pdf]

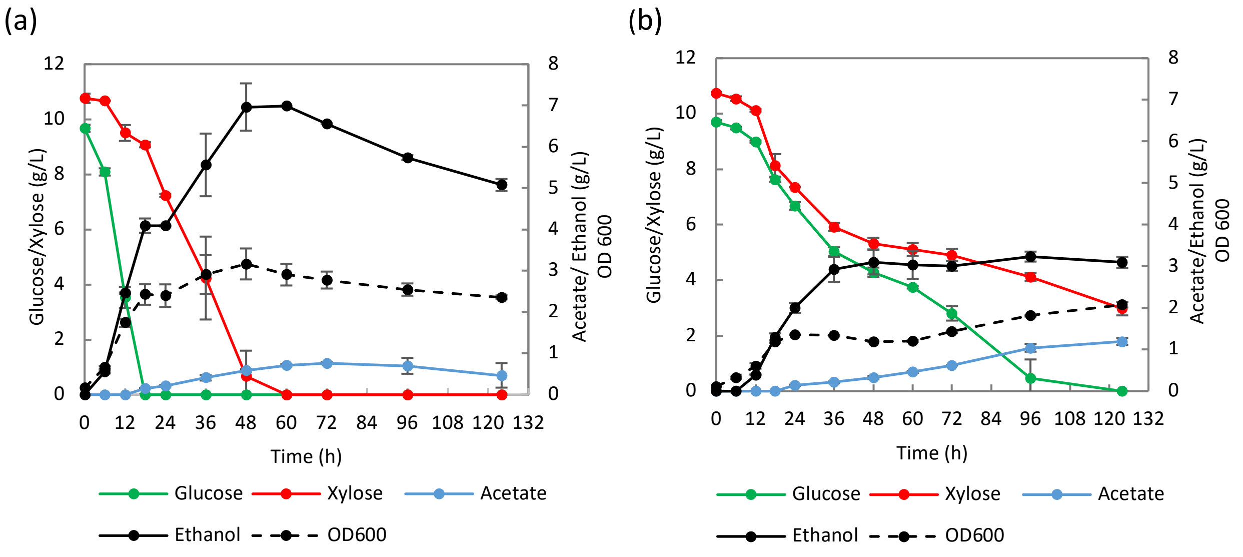

Fig. S1: Sugar consumption, ethanol production and growth profile. Strain SSK42 (a) and SCD00 (b). Growth profile is represented by OD 600.

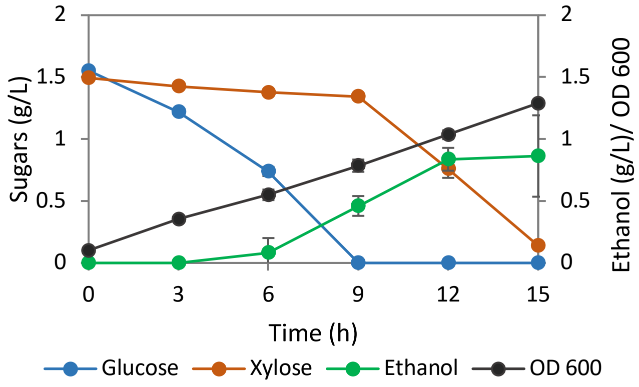

Fig. S2: Sugar consumption, ethanol production and growth profile of strain SCD1200 that has been evolved on xylose and glucose in sequence. Sugars concentrations are plotted on left y-axis while ethanol and optical density are plotted on right y-axis. Optical density represents the growth profile. Data represents the average of two biological replicates.

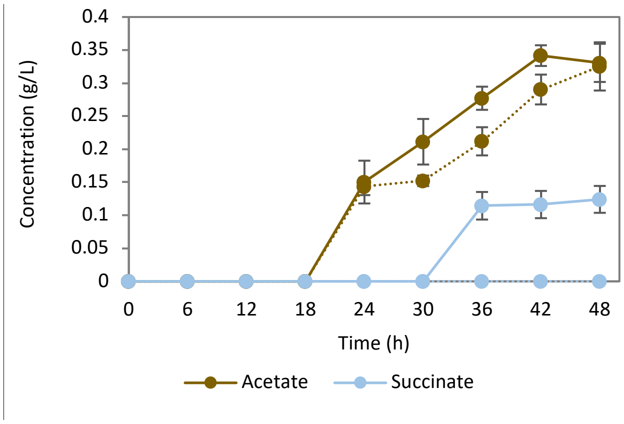

Fig. S3: Metabolite production profiles of strain SCD00 and SCD78. Solid lines represents the strain SCD78 while dotted lines represents the SCD00 strain.

Table S1: Differential gene expression of strain SCD00 vs SSK42

Protein symbol	Name	Log_2fold change	P values
AspA	aspartate ammonia-lyase	6.640	0.000070310
MglB	methyl-galactoside transporter subunit	6.205	0.000102261
PspA	regulatory protein for phage-shock-protein operon	5.156	0.000299718
AraA	L-arabinose isomerase	5.097	0.000552007
Lon	DNA-binding ATP-dependent protease La	4.711	0.000934025
YcbG	hypothetical protein	4.345	0.014679453
GlpK	glycerol kinase	4.116	0.001969458
FlgH	flagellar L-ring protein precursor H	4.046	0.006359024
NuoB	NADH dehydrogenase subunit B	3.992	0.002087669
RbsD	predicted cytoplasmic sugar-binding protein	3.988	0.00210928
FlgM	anti-sigma factor for FliA (sigma 28)	3.968	0.004532581
YjiM	predicted 2-hydroxyglutaryl-CoA dehydratase	3.922	0.001424113
YghZ	aldo-keto reductase	3.891	0.001221941
YebE	hypothetical protein	3.856	0.010470693
ECB_02650	D-ribose-binding periplasmic protein	3.832	0.002115122
RbsB	D-ribose transporter subunit	3.814	0.001968329
RbsA	fused D-ribose transporter subunits of ABC superfamily: ATP-binding components	3.722	0.00343718
AldA	aldehyde dehydrogenase A, NAD-linked	3.668	0.002972645
NuoI	NADH dehydrogenase subunit I	3.580	0.020995499
SucC	succinyl-CoA synthetase subunit beta	3.556	0.007450293
YajC	preprotein translocase subunit YajC	3.484	0.010946762
DacB	D-alanyl-D-alanine carboxypeptidase	3.340	0.025570519
XylA	xylose isomerase	3.253	0.005479046
YdcL	predicted lipoprotein	3.250	0.049735403
SdhA	succinate dehydrogenase flavoprotein subunit	3.197	0.003465327
SdaA	L-serine deaminase I	3.129	0.016945264
YgiM	predicted signal transduction protein (SH3 domain)	2.991	0.006964972
PckA	phosphoenolpyruvate carboxykinase	2.834	0.007512155
NuoE	NADH dehydrogenase subunit E	2.810	0.015638053
XylB	xylulokinase	2.808	0.010564481
SucB	dihydrolipoamide acetyltransferase	2.718	0.010971427
SdhB	succinate dehydrogenase, FeS subunit	2.703	0.010689945
FumA	fumarate hydratase (fumarase A), aerobic Class I	2.702	0.012591306
PpsA	phosphoenolpyruvate synthase	2.691	0.00914812
SucD	succinyl-CoA synthetase subunit alpha	2.650	0.008481498
AceA	isocitrate lyase	2.632	0.020727705
HslV	ATP-dependent protease peptidase subunit	2.593	0.018465653
RbsK	ribokinase	2.589	0.022935044

CpdB	bifunctional 2',3'-cyclic nucleotide 2'-phosphodiesterase/3'-nucleotidase periplasmic precursor protein	2.580	0.01207305
YegU	predicted hydrolase	2.543	0.020410434
Agp	glucose-1-phosphatase/inositol phosphatase	2.478	0.019942638
FolP	7,8-dihydropteroate synthase	2.463	0.026055579
SfsA	sugar fermentation stimulation protein A	2.414	0.027854813
PepB	aminopeptidase B	2.403	0.013116877
Gst	glutathionine S-transferase	2.377	0.016223205
TolC	outer membrane channel precursor protein	2.368	0.016596473
MiaA	tRNA delta(2)-isopentenylpyrophosphate transferase	2.327	0.021855534
TalB	transaldolase B	2.304	0.023031901
YdbC	predicted oxidoreductase, NAD(P)-binding	2.275	0.0332909
SucA	alpha-ketoglutarate decarboxylase	2.249	0.018004303
GroEL	chaperonin GroEL	2.213	0.021321388
HybC	hydrogenase 2, large subunit	2.191	0.021019659
YdhH	anhydro-N-acetylmuramic acid kinase	2.181	0.036058409
YqiC	hypothetical protein	2.077	0.042980858
LysS	lysine tRNA synthetase, constitutive	2.019	0.028119157
HflX	predicted GTPase	-2.014	0.041550361
RplD	50S ribosomal protein L4	-2.043	0.029300296
RplP	50S ribosomal protein L16	-2.057	0.039110656
LysU	lysine tRNA synthetase, inducible	-2.062	0.036229669
YceD	hypothetical protein	-2.076	0.040366807
GltI	glutamate and aspartate transporter subunit	-2.081	0.033674595
YhbH	predicted ribosome-associated, sigma 54 modulation protein	-2.128	0.042062014
Rne	fused ribonucleaseE: endoribonuclease/RNA-binding protein/RNA degradosome binding protein	-2.189	0.021154308
RpmE	50S ribosomal subunit protein L31	-2.204	0.019350119
GcvP	glycine dehydrogenase	-2.230	0.019921568
RplR	50S ribosomal protein L18	-2.248	0.021513019
YidA	predicted hydrolase	-2.253	0.033289114
UvrA	excinuclease ABC subunit A	-2.291	0.023073982
YjeB	predicted DNA-binding transcriptional regulator	-2.345	0.036785727
TrpD	bifunctional indole-3-glycerol-phosphate synthase/anthranilate phosphoribosyltransferase	-2.379	0.022138686
FruA	fused fructose-specific PTS enzymes: IIBcomponent/IIC components	-2.426	0.037538629
SrmB	ATP-dependent RNA helicase	-2.446	0.034485974
YjfH	23S rRNA (Gm2251)-methyltransferase	-2.500	0.035648948
Dld	D-lactate dehydrogenase, FAD-binding, NADH independent	-2.507	0.042648822
YicH	hypothetical protein	-2.513	0.020963834
YahK	predicted oxidoreductase, Zn-dependent and NAD(P)-binding	-2.517	0.014197307
RpsP	30S ribosomal protein S16	-2.537	0.010929715
AdhE	alcohol dehydrogenase	-2.580	0.014511012
RpsF	30S ribosomal protein S6	-2.580	0.042946556

RplB	50S ribosomal protein L2	-2.597	0.038186999
YdgJ	predicted oxidoreductase	-2.619	0.04612702
YiiT	stress-induced protein	-2.639	0.036966602
GltB	glutamate synthase, large subunit	-2.712	0.011967592
SpeA	arginine decarboxylase	-2.722	0.013083404
Cfa	cyclopropane fatty acyl phospholipid synthase (unsaturated-phospholipid methyltransferase)	-2.740	0.027494086
MutS	DNA mismatch repair protein	-2.831	0.033083762
GlnA	glutamine synthetase	-2.857	0.007958092
YrbH	D-arabinose 5-phosphate isomerase	-2.969	0.035355664
DppD	dipeptide transporter	-2.982	0.028226843
NrdA	ribonucleotide-diphosphate reductase alpha subunit	-3.013	0.029336957
ArcB	hybrid sensory histidine kinase in two-component regulatory system with ArcA	-3.025	0.004660799
Rnr	exoribonuclease R, RNase R	-3.047	0.007182885
FruB	fused fructose-specific PTS enzymes: IIA component/HPr component	-3.147	0.011020542
YfiF	predicted methyltransferase	-3.302	0.008795573
RpsO	30S ribosomal protein S15	-3.337	0.013210312
ManZ	mannose-specific enzyme IID component of PTS	-3.972	0.001714941
ArgI	ornithine carbamoyltransferase 1	-3.973	0.00105122
SelB	selenocysteinyl-tRNA-specific translation factor	-4.084	0.001429008
PepA	leucyl aminopeptidase	-4.159	0.004511832

Table S2: Differential gene expression of strain SCD78 vs SCD00

Protein symbol	**Name**	**Log_2fold change**	**P values**
SelB	selenocysteinyl-tRNA-specific translation factor	5.902	0.000527
SfcA	malate dehydrogenase, (decarboxylating, NAD-requiring) (malic enzyme)	5.233	0.00062
YgiF	predicted adenylate cyclase	4.085	0.000444
ArcB	hybrid sensory histidine kinase in two-component regulatory system with ArcA	4.076	0.001506
RncS	ribonuclease III	4.050	0.016104
MetC	cystathionine beta-lyase	4.007	0.00186
SspB	ClpXP protease specificity-enhancing factor	3.723	0.001559
CspA	major cold shock protein	3.722	0.005427
YgfB	hypothetical protein	3.618	0.001872
MgsA	methylglyoxal synthase	3.379	0.002696
MogA	molybdenum cofactor biosynthesis protein	3.162	0.014286
YdcG	glucan biosynthesis protein, periplasmic	3.128	0.017582
UdhA	soluble pyridine nucleotide transhydrogenase	3.126	0.003489
Usg	hypothetical protein	3.117	0.026361
FruB	fused fructose-specific PTS enzymes: IIA component/HPr component	3.100	0.015079
PepA	leucyl aminopeptidase	3.082	0.029071
AldA	aldehyde dehydrogenase A, NAD-linked	3.071	0.001643
YjbR	hypothetical protein	3.047	0.032427
LolA	outer-membrane lipoprotein carrier protein precursor	2.942	0.014316
YicH	hypothetical protein	2.890	0.01457
Rnr	exoribonuclease R, RNase R	2.884	0.006658
ManZ	mannose-specific enzyme IID component of PTS	2.862	0.020367
Nfi	endonuclease V	2.798	0.016018
SrmB	ATP-dependent RNA helicase	2.710	0.027361
YciM	hypothetical protein	2.681	0.00328
Ppk	polyphosphate kinase	2.510	0.010843
YcdW	2-ketoacid reductase	2.463	0.036535
PepE	peptidase E	2.437	0.039302
YjiM	predicted 2-hydroxyglutaryl-CoA dehydratase	2.411	0.004571
FruK	phosphofructokinase	2.392	0.047225
YceH	hypothetical protein	2.374	0.008079
PurF	amidophosphoribosyltransferase	2.373	0.009246
YiiT	stress-induced protein	2.317	0.012055
MetH	B12-dependent methionine synthase	2.275	0.008852
MtlD	mannitol-1-phosphate 5-dehydrogenase	2.230	0.037359
YdaA	stress-induced protein	2.207	0.016726
Tsx	nucleoside channel, receptor of phage T6 and colicin K	2.146	0.016476
LplA	lipoate-protein ligase A	2.125	0.015527

Psd	phosphatidylserine decarboxylase	2.121	0.045306
UvrA	excinuclease ABC subunit A	2.102	0.0154
IscA	iron-sulfur cluster assembly protein	2.070	0.02161
HisI	bifunctional phosphoribosyl-AMP cyclohydrolase/phosphoribosyl-ATP pyrophosphatase protein	2.069	0.023958
Dps	DNA protection protein	2.054	0.028899
RpsH	30S ribosomal protein S8	2.036	0.016841
TyrB	tyrosine aminotransferase, tyrosine-repressible, PLP-dependent	2.034	0.009717
DamX	Cell division protein	-2.013	0.011943
NudG	pyrimidine (deoxy)nucleoside triphosphate pyrophosphohydrolase	-2.022	0.020762
TrpR	Trp operon repressor	-2.037	0.0411
BaeR	DNA-binding response regulator in two-component regulatory system with BaeS	-2.045	0.015964
PrfC	peptide chain release factor 3	-2.079	0.011169
YqiC	Ubiquinone biosynthesis accessory factor	-2.092	0.024605
YicC	hypothetical protein	-2.101	0.047441
PepT	peptidase T	-2.122	0.046547
YeaY	predicted lipoprotein	-2.153	0.032957
YbaK	Cys-tRNA^{Pro} and Cys-tRNA^{Cys} deacylase	-2.192	0.005973
DjlA	Dna-J like membrane chaperone protein	-2.226	0.017277
YcfP	hypothetical protein	-2.230	0.022309
PyrI	aspartate carbamoyltransferase regulatory subunit	-2.231	0.005762
YdhD	glutaredoxin 4	-2.273	0.037809
YbiB	nonspecific DNA-binding protein	-2.275	0.013307
YecJ	hypothetical protein	-2.298	0.014323
YibN	predicted rhodanese-related sulfurtransferase	-2.310	0.016684
DacB	D-alanyl-D-alanine carboxypeptidase	-2.370	0.014545
GpsA	NAD(P)H-dependent glycerol-3-phosphate dehydrogenase	-2.402	0.006948
YtfP	hypothetical protein	-2.451	0.011767
YggB	mechanosensitive channel	-2.516	0.003646
PrfA	peptide chain release factor 1	-2.528	0.004231
GnsB	predicted protein	-2.597	0.007092
MurD	UDP-N-acetylmuramoyl-L-alanyl-D-glutamatesynthetase	-2.674	0.01774
YgjD	O-sialoglycoprotein endopeptidase	-2.736	0.043391
Lrp	DNA-binding transcriptional dual regulator, leucine-binding	-2.761	0.021767
YhgI	predicted gluconate transport associated protein	-2.812	0.018464
YdcL	predicted lipoprotein	-2.874	0.004119
CydC	fused cysteine transporter subunits of ABC superfamily: membrane component/ATP-binding component	-3.069	0.036828
YadF	carbonic anhydrase	-3.102	0.008802
TldD	predicted peptidase	-3.169	0.001251
YegU	predicted hydrolase	-3.213	0.005911
Lpp	murein lipoprotein	-3.732	0.037601

RbsB	D-ribose transporter subunit	-4.123	0.003029
RbsK	ribokinase	-4.466	0.002476
PspA	regulatory protein for phage-shock-protein operon	-5.515	0.000124
